# A One Health Evaluation of the Southern African Centre for Infectious Disease Surveillance

**DOI:** 10.3389/fvets.2018.00033

**Published:** 2018-03-16

**Authors:** Marie C. E. Hanin, Kevin Queenan, Sara Savic, Esron Karimuribo, Simon R. Rüegg, Barbara Häsler

**Affiliations:** ^1^Department of Pathobiology and Population Sciences, Veterinary Epidemiology Economics and Public Health Group, Royal Veterinary College, London, United Kingdom; ^2^Scientific Veterinary Institute Novi Sad, Novi Sad, Serbia; ^3^Sokoine University of Agriculture, Morogoro, Tanzania; ^4^Vetsuisse Faculty, University of Zurich, Zurich, Switzerland

**Keywords:** Southern African Centre for Infectious Disease Surveillance, One Health, evaluation, capacity, surveillance

## Abstract

Rooted in the recognition that emerging infectious diseases occur at the interface of human, animal, and ecosystem health, the Southern African Centre for Infectious Disease Surveillance (SACIDS) initiative aims to promote a trans-sectoral approach to address better infectious disease risk management in five countries of the Southern African Development Community. Nine years after SACIDS’ inception, this study aimed to evaluate the program by applying a One Health (OH) evaluation framework developed by the Network for Evaluation of One Health (NEOH). The evaluation included a description of the context and the initiative, illustration of the theory of change, identification of outputs and outcomes, and assessment of the One Healthness. The latter is the sum of characteristics that defines an integrated approach and includes OH thinking, OH planning, OH working, sharing infrastructure, learning infrastructure, and systemic organization. The protocols made available by NEOH were used to develop data collection protocols and identify the study design. The framework relies on a mixed methods approach by combining a descriptive and qualitative assessment with a semi-quantitative evaluation (scoring). Data for the analysis were gathered during a document review, in group and individual interviews and in an online survey. Operational aspects (i.e., OH thinking, planning, and working) were found to be balanced overall with the highest score in the planning dimension, whereas the infrastructure (learning infrastructure, systemic organization, and sharing infrastructure) was high for the first two dimensions, but low for sharing. The OH index calculated was 0.359, and the OH ratio calculated was 1.495. The program was praised for its great innovative energy in a difficult landscape dominated by poor infrastructure and its ability to create awareness for OH and enthuse people for the concept; training of people and networking. Shortcomings were identified regarding the balance of contributions, funds and activities across member countries in the South, lack of data sharing, unequal allocation of resources, top-down management structures, and limited horizontal collaboration. Despite these challenges, SACIDS is perceived to be an effective agent in tackling infectious diseases in an integrated manner.

## Introduction

Emerging infectious diseases have always been, and are still today, a major burden for human populations (1). This burden is particularly high in low- and middle-income countries (2), and Africa is considered the continent to suffer the most from infectious diseases (3) and to have the least capacities to control them. More than 60% of human emerging infectious diseases are zoonotic, meaning they can be transmitted from animals to humans or *vice versa* (4). Our modern societies and new ways of life are changing the dynamics of zoonotic diseases transmission (5). Deforestation, international trade, hunting, or ecotourism are all factors that increase the likelihood of contacts between humans and wildlife facilitating potential spillovers of zoonotic pathogens from a wildlife reservoir to humans (6, 7). In addition, pathogens’ and vectors’ transmission cycles and ability to spread and adapt depend on the environment in which they evolve. Tackling emerging infectious diseases requires the study of environmental factors that lead to the modification of ecosystems, such as climate change, biodiversity loss, or other anthropogenic causes (1, 8).

Rooted in this understanding that emerging infectious diseases occur at the interface of human health, animal health, and ecosystem health (4), the Southern African Centre for Infectious Disease Surveillance (SACIDS) initiative aims to promote a trans-sectoral approach to address better infectious disease risk management in Southern African countries. SACIDS’ vision was outlined in 2008 after a series of meetings and workshops held between five Southern African Development Community (SADC) countries and international partners. As defined at the inception of the initiative, SACIDS’ mission is to “harness innovation in science and technology to improve Southern Africa’s capacity (including human, financial, and physical) to detect, identify, and monitor infectious diseases of humans, animals, plants, and their interactions to better manage the risk posed by them” (9). The creation of SACIDS was in line with the gradual recognition of the One Health (OH) concept, which gained momentum at the beginning of the twenty-first century following the highly pathogenic avian influenza outbreaks (10, 11). Although it is hard to reduce this transdisciplinary approach to one definition, the description made by the Food and Agriculture Organization of the United Nations gives a good idea of the OH principles: “A collaborative, international, cross-sectorial, multidisciplinary mechanism to address threats and reduce risks of detrimental infectious diseases at the animal–human–ecosystem interface” (11).

Nine years after its establishment, the SACIDS program benefited from an external evaluation. The added value arising from integration and its transdisciplinary approach was highlighted in the evaluation report (12). However, OH is still a relatively new concept, and few studies have been implemented to evaluate systematically OH initiatives (13). The Cooperation on Science and Technology Action Network for Evaluation of One Health (NEOH, http://neoh.onehealthglobal.net/, accessed April 17, 2017), is working toward the establishment of a science-based evaluation protocol to enable quantitative and qualitative evaluation of various OH activities. This protocol aims to provide an assessment of the strength of an OH initiative by confronting the OH process characteristics of operations (thinking, planning, and working) and supporting infrastructure (systemic organization, learning, and sharing) with achieved changes and comparing it to outcomes generated by the initiative (14).

With a focus on the surveillance of infectious diseases, this study aimed to evaluate the OH capacity building program SACIDS by using the framework developed by NEOH. The data used for the evaluation were collected from different SACIDS stakeholders and actors in Tanzania, Zambia, Mozambique, Democratic Republic of Congo (DRC), and the UK using different data collection approaches. We present the context in which SACIDS evolves, describe the theory of change and the outcomes of the initiative and the assessment of the different OH dimensions of SACIDS.

## Methods

### General Overview

The evaluation questions for this study were as follows: *What is the context within which SACIDS operates and how does it link to this context? What is the SACIDS theory of change? What are the outputs and impact achieved? How can the different OH dimensions in SACIDS be characterized? Which elements were particularly strong and which could be improved to ensure that longer-term impacts can be realized?*

The Southern African Centre for Infectious Disease Surveillance was interested in supporting this evaluation to get an assessment of its OH dimensions and to understand which elements were working well and which ones could potentially be improved in the future. To achieve this, we conducted a process evaluation that was based on the One Healthness (OHness) framework from NEOH (14) and described the outcomes achieved by the program.

The protocols made available by NEOH (14) were used to develop data collection protocols and identify the study design. The NEOH framework relies on a mixed methods approach by combining a descriptive and qualitative assessment with a semi-quantitative evaluation (scoring) for the evaluation of OHness with an OH index (OHI), while including conventional metrics on the outcomes’ side. The descriptive part is based on systems thinking.

### Data Collection

The data collection methods included a document review, face-to-face group and individual interviews, phone interviews, and an online survey. Data were collected between March and July 2017 during visits to Zambia and Tanzania as well as interviews conducted from the UK.

Documents for the document review were requested and obtained from SACIDS Executive Director and secretariat. They were asked to share any documentation including proposals, reports, presentations, peer-reviewed and lay publications that described the SACIDS program and journey from its inception to the end of the Phase 1 funding (i.e., 2008–2017).

Following the document review, two questionnaires were developed to gather insights and perspectives from SACIDS members. The first one was a questionnaire to be administered in face-to-face interviews to collect data on OH data sharing infrastructures, OH planning, and OH working components of SACIDS. The main goal of this questionnaire was to enable discussion between the participants and to capture the main issues on which people agreed or disagreed. The detailed question guide can be found in Table S1 in Supplementary Material. Target respondents were SACIDS members from all SACIDS active countries (i.e., Tanzania, Zambia, the DRC, Mozambique, South Africa, and the UK) with long-standing SACIDS experience, i.e., people who had been part of the program for at least 4 years. Moreover, different levels of seniority were aimed for with invited participants spanning postgraduate students, postdocs, community of practice (CoP) leaders, initiators, advisors, and management board members. The questionnaire was used in two group interviews in Morogoro, Tanzania with five SACIDS members, and in Lusaka, Zambia, with six SACIDS members. Moreover, individual interviews were conducted with the SACIDS Executive Director and a SACIDS smart partner in the UK (smart partnerships in SACIDS are partnerships with institutions that can provide specific input and expertise to the program) plus a representative from Mozambique. No partner from South Africa was interviewed due to resource constraints. The decision to interview people in either group or individual interviews, respectively, was made solely on practical considerations and the availability of people. People who could not be met personally were interviewed using the online application Skype 7.52; one person in the DRC and one person in the UK were interviewed in this way. The complete question guide for the personal interviews was too long to apply in the same detail to all respondents. Consequently, the interviewers allowed more time for the questions that the respondents seemed to be most knowledgeable about. Some of the questions on the management of SACIDS were skipped when participants (mostly MSc or PhD students) claimed that they did not know how exactly SACIDS was managed. When there was an unresolved disagreement in a group interview, the interviewer noted down both opinions.

The second questionnaire was an online survey in the software Google Forms to collect data on the dimensions of OH learning and OH thinking (Table S2 in Supplementary Material). The survey was widely distributed in the SACIDS community based on an email list shared by the SACIDS secretariat and followed-up by two personal reminders by email. The target respondents were the same as for the personal interviews. Apart from the online approach, the same questionnaire was used with individual SACIDS members, namely, four in Tanzania, eight in Zambia, and one in the UK.

All face-to-face interviews were held in English by the first and last author, respectively. Written notes were taken during the interviews and summarized afterward. Ethical approval for the interviews and the online survey was sought from the Royal Veterinary College (RVC) and was granted by its Social Sciences Research Ethical Review Board, number URN SR2017-1002.

### Data Analysis

The context, the description of the initiative, and the theory of change were derived from the document review and refined based on information gathered during the interviews with SACIDS members. Outputs and outcomes were identified during the document review and complemented by information shared by respondents during the interview and surveys. The assessment of the outputs and outcomes was descriptive only.

After the data collection was completed, the extent of the OHness was assessed by following the recommendations developed in the NEOH tools (14); all dimensions, the information collected for each element, and the scoring can be found in Table S3 in Supplementary Material. The OHness is a sum of characteristics that defines an integrated approach and includes OH thinking, OH planning, OH working, sharing infrastructure, learning infrastructure, and systemic organization (14). The understanding and capacity to use the tools was enhanced by training future evaluators in July 2016, organized by NEOH.

Metrics are described and detailed in the NEOH tools (14), and a short summary of each element is provided below. Every question was scored based on a detailed explanation of the arguments gathered during the various interviews, and the reasoning for the score was presented. Each OH characteristic was described by a final score, summarizing the question-specific scores. In the end, an OH spider diagram was constructed, and an OHI and OH ratio (OHR) calculated using the equations presented in Ref. (14).

The data collection and assessment were mainly conducted by the first and last author, respectively, based on information shared by SACIDS members and the documents reviewed. Where there was a difference in the scoring, they discussed the discrepancy, presented their arguments, and agreed on a score. A general review of the evaluation was completed by the coauthors, all external to SACIDS, but with the Tanzanian coauthor closely collaborating with the initiative.

#### OH Thinking

Based on the assumption that the integration of human, animal, and ecosystem health requires systems thinking and the consideration of multiple dimensions, feedback loops, and interconnectedness (14), the questions probing for system thinking relied on the match between the dimensions of the initiative and its context as well as key elements of systems thinking. Particular attention was given to the scales in different dimensions, and that the initiative reflects the context in which it operates. The score given for the quantitative evaluation of SACIDS OH thinking resulted from the mean of six different categories: (1) the balance of consideration of the different dimensions by the initiative, (2) the match between the initiative and its context, (3) the initiative’s integrated approach to health, (4) the system features targeted by the initiative, (5) the initiative’s considerations of sustainability and socioecological factors, and (6) the consideration of different perspectives and factors that impact on the theory of change.

#### OH Planning

The planning score is built on the assumption that careful planning of tasks and activities in line with the initiative’s objectives and goals in an OH way necessitates careful and balanced allocation of resources to all tasks and objectives under consideration of the integrated nature of the program. Consequently, this score includes the description of (1) common aims, (2) stakeholder and actor engagement, (3) self-assessment and plan revisions; and (4) matching of planning and resources for all objectives.

#### OH Working

Interdisciplinary collaboration brings together people with different skills, expertise, experience, backgrounds, and often from varying epistemologies with the aim to tackle complex problems with a high societal stake that require an understanding of the human behavior (15–17). In OH, interdisciplinarity has merged with a participatory approach in the form of transdisciplinarity (17), which relies on appropriate leadership and management that is visionary, supportive, and engaging (17, 18) to create a strategic dialog, shared decision-making, and non-hierarchical relationships and allows for self-reflection, flexibility, and recursiveness (17, 19–21). These considerations were the basis for the OH working assessment, which considered (1) the breadth of the initiative, (2) collaboration, (3) transdisciplinary balance, (4) cultural and social balance, and (5) flexibility and adaptation.

#### Sharing Infrastructure

In a broad sense, data and information sharing is a science catalyzer (22). However, data and shared information can also stimulate progress in both mandatory and voluntary interventions, surveillance and control programs, e.g., when used for certification, open-access reporting opportunities or surveillance, and benchmarking (23). A central benefit of data sharing stems from analyzing data more comprehensively and developing further knowledge and information. In the NEOH sharing tool, the following elements were considered: (1) general information and awareness of sharing, (2) data and information sharing, (3) methods and results sharing, and (4) institutional memory/resilience.

#### Learning

Learning, a change in cognition, potential behavior, or actual behavior through better knowledge and understanding, can be achieved at the individual, group, and organizational levels (24), strongly influenced by the interplay between them (25). In other words, they work together and influence each other (26). The assessment of learning was done using the individual questionnaires. Five categories were evaluated, each one being composed of two to three questions: (1) adaptive and generative individual learning, (2) adaptive and generative team learning, (3) adaptive and generative organizational learning, (4) direct learning environment supportive of adaptive and generative learning, and (5) general learning environment supportive of adaptive and generative learning. For each of the 13 questions, the score given was the mean of the answers obtained in the individual questionnaires. Then, within a category, each question was weighted depending on its importance (questions relating to generative learning had more weight than questions relating to adaptive learning, which had more weights than the ones relating to basic learning).

#### Systemic Organization

In many complex settings, change-oriented leadership can overcome rigid conventions, norms, and traditions by targeting leverage points in systems to modify behavior. In OH, there is also the challenge that collaboration may be dominated by one discipline, which can reduce commitment, interest, and motivation. A solution is shared/distributed leadership and governance (27) to promote engagement of all disciplines and unlock creative potential, competence, and innovation. Consequently, the selection of questions for the scoring of the systemic organization of OH initiatives involved (1) team structures, (2) social and leaderships structures and skills, (3) competence, and (4) focus and innovation.

#### OHI and OHR

Once all scores for the six dimensions were available, the six assessments were illustrated in a spider diagram, in which each assessment was represented by a spoke. The diagram depicts the operational aspects “OH thinking,” “OH planning,” and “OH working” opposed to the infrastructure for “learning,” “sharing,” and “systemic organization.” Based on this, the OHI is computed by calculating the proportion of the surface of the hexagon covered, while the OHR is the relation of the surface covered in the top left of the diagonal to the one in the lower right (14).

## Evaluation Results

### Definition of the Context and the Initiative

#### General Overview

The Southern African Centre for Infectious Disease Surveillance is a regional consortium established and operating in Tanzania, Zambia, Mozambique, DRC, and South Africa. In addition to these five countries, SACIDS also engaged smart partnerships with institutions in Kenya and in the UK.

Several drivers motivated the creation of SACIDS and the willingness to adopt an OH approach. The main argument behind SACIDS inception was the understanding that zoonotic diseases increase both the human health burden and losses of animal production. The synergy of the health and economic consequences of such diseases, amplified by the poor governance systems and civil instabilities, the lack of participatory health policies, of personnel and of resources and the need for adequate leadership, exacerbates poverty (3, 28, 29). All of these factors call for a syndemic approach to tackle infectious diseases (30) through better collaboration between human health, animal health, environmental health, and socioeconomic sectors.

In each African participating country, several institutions from the academic, government, or research sectors participate in SACIDS activities and engage various actors with human health or animal health backgrounds. Following an OH approach, the main objectives of SACIDS are to enhance institutional capacities for African-led research, to promote collaboration between veterinary, medical, and other sectors involved, and to enable better sharing of information and resources between individuals and institutions at a national, regional, and international level. To reach these objectives, SACIDS’ strategy is concentrated on five pillars, described as follows: (1) to enhance the capacity of institutions for the detection, identification, and monitoring of infectious diseases of both humans and animals, in a “one medicine” framework, (2) to enhance biosafety and quality management (BQM), (3) to enhance skills through taught and distance-learning programs, (4) to enhance information and communication technologies (ICT) to support learning and disease surveillance systems, and (5) to enhance skills through research. By promoting joint efforts in education, communication, research, and disease surveillance, SACIDS is a pioneer initiative in the adoption and application of OH principles for the surveillance of infectious diseases in Southern Africa.

#### Description and Visual Representation of the Context

The Southern African Centre for Infectious Disease Surveillance is an initiative that exists in five countries of the SADC,^1^ which has been working toward the establishment of “economic development, peace and security, and growth” since the 1980s and aimed to “enhance the standard and quality of life of the people of Southern Africa and support the socially disadvantaged through regional integration” (31). Before the establishment of SACIDS, the SADC had already promoted some linkages and network building at a regional level, which was one of the reasons to select only SADC countries to build SACIDS.

The political context in which SACIDS evolves is a legacy of the post-colonial era. The whole process of political change in Africa took decades from the 1950s to the 1990s, and Southern African countries were late in this transition. This has been an obstacle to the implementation of disease control programs in these countries. Political instabilities remain a challenge to long term policy planning, as in the DRC where the rapid turnover of governments makes it difficult to create solid links between individuals or institutions. Regarding capacity for the surveillance of infectious diseases, only South Africa had adequate tools and expertise to monitor and control human and animal emerging infectious diseases. The country had the ability, the infrastructures, and the capacity to guide the other countries that were too weak to match globally agreed standards.

**Figure 1:**
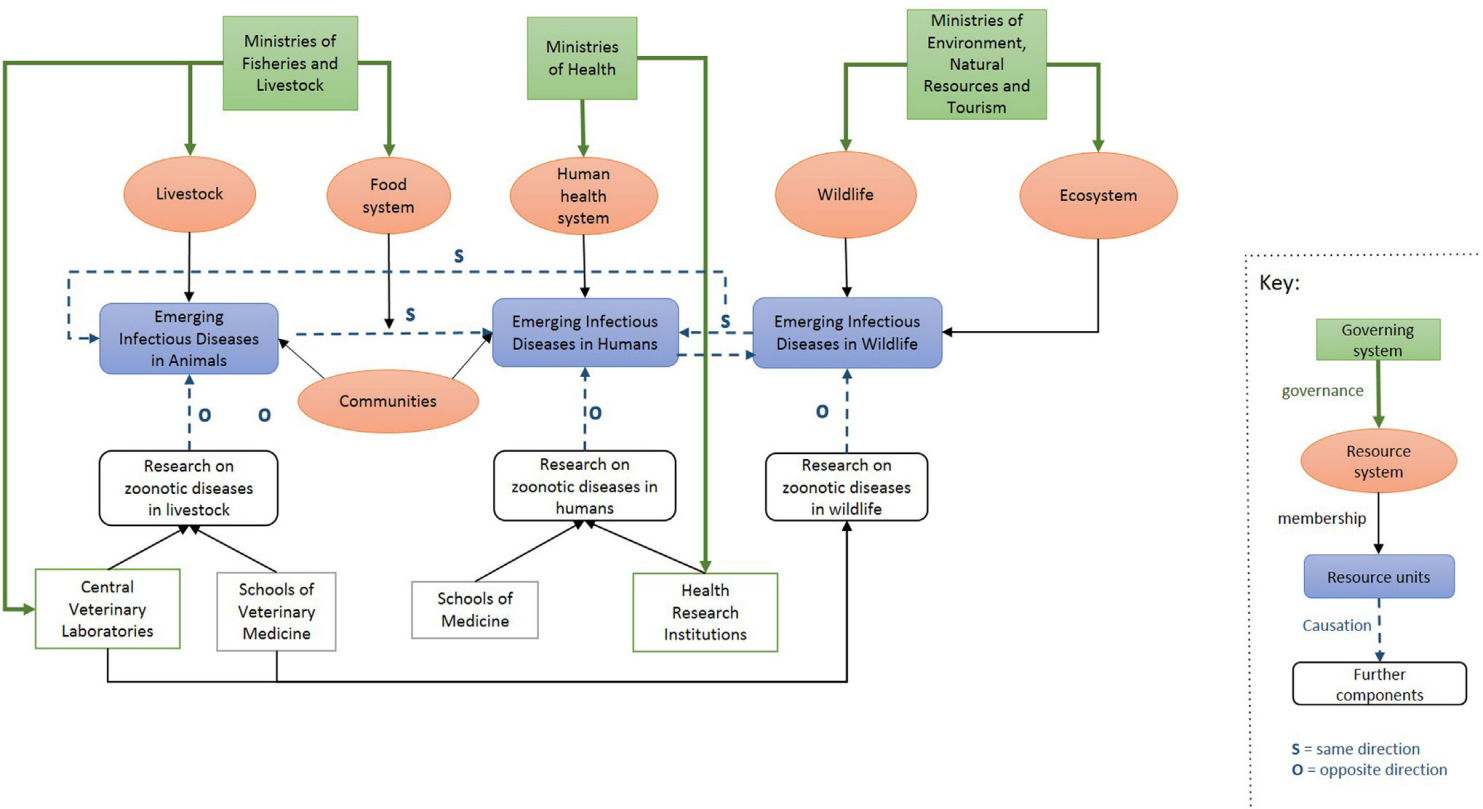
Overview of the context within which Southern African Centre for Infectious Disease Surveillance operates in Southern African countries.

Figure 1 gives an overview of the context within which SACIDS was developed. This provides a general impression of the interactions happening at a national level in the participating countries between human health, animal health, and environmental sectors and between governmental and academic institutions. Although each country has its own specificities, the overall academic and government structures used to shape SACIDS at the national level were found to be similar: a ministry of health, a ministry of agriculture/livestock, a government structure for the environment and/or the wildlife, national institutes for research in human and animal health, and universities with both schools of human medicine and veterinary medicine. Tanzania and Zambia already had some postgraduate programs at their universities and had started to create some connections between veterinarians and human doctors. In Mozambique and DRC, a crucial lack of institutional capacity was noted, with no scientific PhD programs in place in Mozambique and no veterinary school in Kinshasa, the capital city of DRC.

#### Description of the OH Initiative in Relation to Its Context

The Southern African Centre for Infectious Disease Surveillance was created as a consortium in 2008 under the guidance of its present Executive Director. It was guided by two international foresight studies that called for action against infectious diseases (32, 33). Hence, it arose from the recognition that a majority of emerging infectious diseases were zoonotic, and that joint efforts from the human health and animal health sectors were necessary to control them effectively. As described in the first evaluation report of SACIDS, the initiative’s main goal is “to enhance institutional capacities for intersectoral research approaches to tackling infectious disease priorities through ‘One-Health’ approaches in both universities and research institutes across southern Africa” (34).

From 2008 to 2016, SACIDS received funding from different organizations including the Wellcome Trust foundation, Google, the Rockefeller Foundation, the African Development Bank, and the International Research Centre. SACIDS was selected by the Wellcome Trust to be one of the seven consortiums that constitute the African Institutions Initiatives, which aim to improve research capacity and African-led research through networked approaches in 18 African countries and 51 institutions. SACIDS resulted in the partnership of five Southern African countries: Tanzania, Zambia, DRC, Mozambique, and South Africa. In total, 18 institutions engaged with SACIDS from the beginning, namely, in Tanzania: Sokoine University of Agriculture (SUA) in Morogoro, Muhimbili University of Health and Allied Sciences in Dar es Salaam, National Institute for Medical Research in Dar es Salaam, and Tanzania Veterinary Laboratory Agency in Dar es Salaam; Mozambique: Eduardo Mondlane University (UEM) in Maputo, Institute of Agricultural Research (Ministry of Agriculture) in Maputo, and National Health Institute (Ministry of Health) in Maputo; DRC: University of Kinshasa, Central Veterinary Laboratory in Kinshasa, and University of Lubumbashi; Zambia: University of Zambia (UNZA) in Lusaka, Central Veterinary Research Institute in Lusaka, and Tropical Diseases Research Institute in Ndola; and South Africa: National Institute for Communicable Diseases in Johannesburg, Veterinary Institute of the Agricultural Research Council in Pretoria, University of Pretoria, and Stellenbosch University. Each country therefore had at least one university, one human health, and one animal health institute engaged with SACIDS, which reflected the consortium’s aim to encourage collaboration between human and animal health sectors and to promote scientific training at a postgraduate level.

The Southern African Centre for Infectious Disease Surveillance also engaged with external partner institutions that are not directly part of SACIDS but which collaborated on some specific research projects, helped on the establishment of training courses and gave external advice to the initiative over time. The main partners are the International Livestock Research Institute, Nairobi, Kenya; the RVC, London, UK; the London School of Hygiene and Tropical Medicine (LSHTM), London, UK; and the London International Development Centre (LIDC), London, UK.

**Figure 2:**
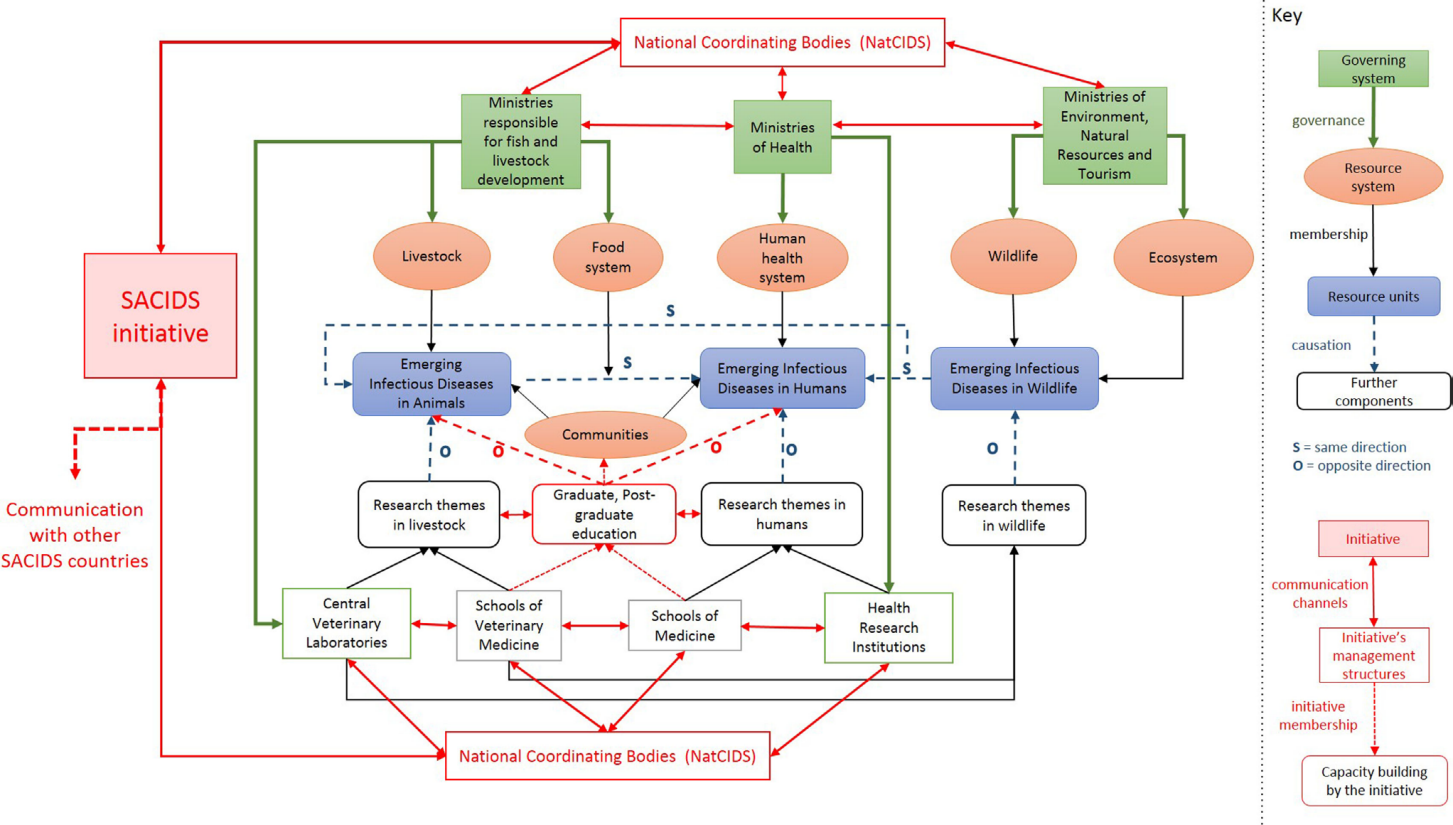
The Southern African Centre for Infectious Disease Surveillance (SACIDS)’ program placed in the context within which it operates.

Figure 2 shows the effect of the SACIDS program within its context. Through the establishment of coordinating structures at the national level, SACIDS created channels of communication between institutions from different sectors and enabled better collaboration between them. SACIDS aimed to tackle the existing silo structure and to create horizontal links between sectors both at the government and academic level. SACIDS also launched postgraduate training classes and encouraged research on emerging and vector-borne diseases, bacterial diseases (including food-borne diseases and antimicrobial resistance), viral diseases of food security importance, and cross-cutting OH sciences, which should not only have a positive effect on the control of emerging infectious diseases in Southern Africa but also create interdisciplinary networks among students—an important foundation for future collaboration between sectors. SACIDS’ secretariat, based in Tanzania, acts as the principal coordinator between the participating countries and with SACIDS’ external partners.

**Figure 3:**
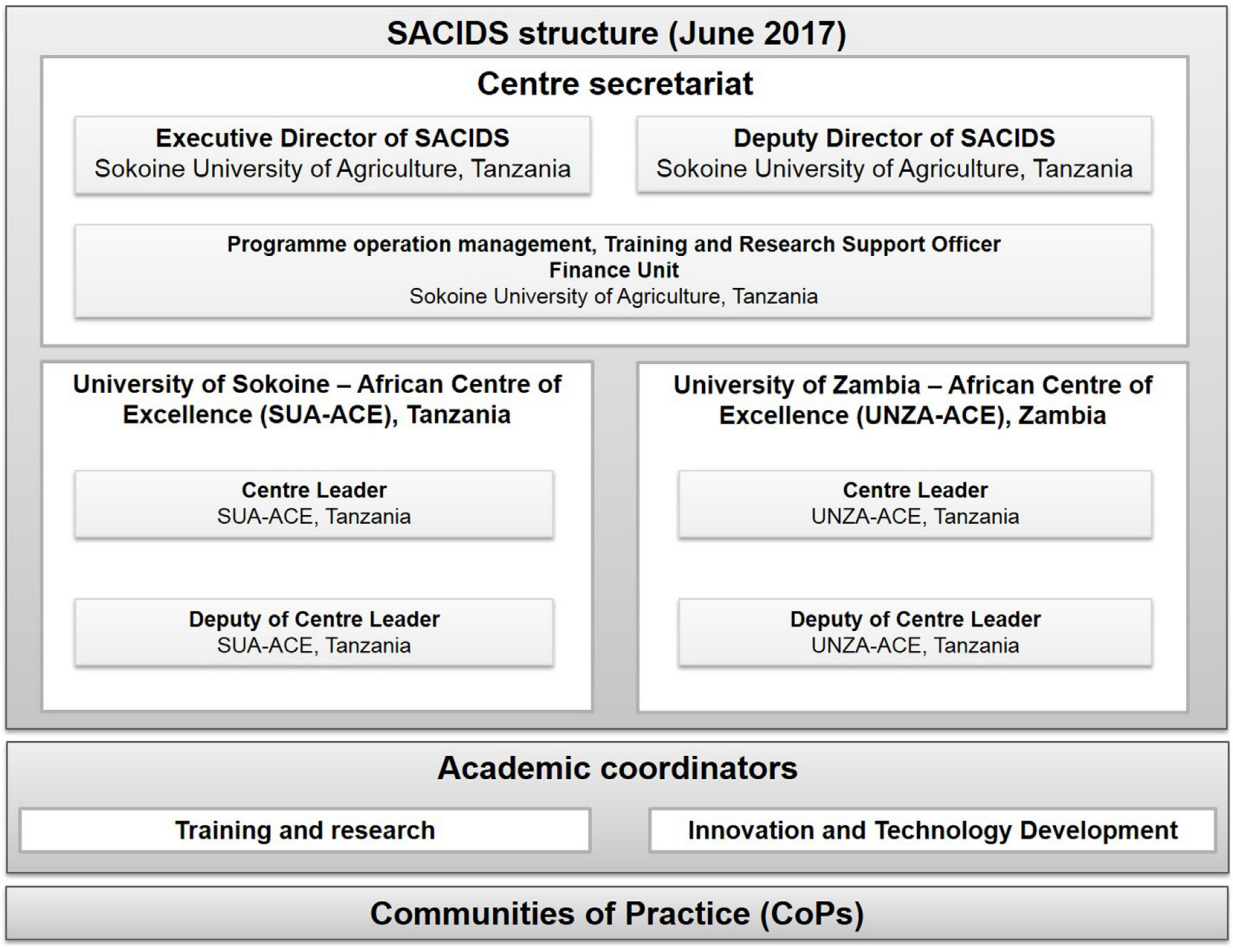
Southern African Centre for Infectious Disease Surveillance (SACIDS)’ structural organization at the regional level.

At the regional level, SACIDS’ management structure is established according to inter-institutional agreements. At the beginning, SACIDS had an Executive Director assisted by two Deputy Directors of which one was responsible for animal health (from Tanzania) and another responsible for human health (from South Africa). Since 2016, through World Bank funding, SACIDS has been running two African Centres of Excellence (ACEs), namely, one in Tanzania at SUA and one in Zambia at the UNZA. Both ACEs report to SACIDS through the SACIDS Governing Board. Each ACE is led by a Centre Leader with a deputy. The Deputy Centre Leader in Tanzania is the same person as the SACIDS Executive Director. Moreover, SACIDS has a secretariat, composed (apart from the Executive Director) of a program operation manager, a “training and research support officer” and a finance unit (Figure 3). The coordination at the academic level is operated by three people: one in charge of the “training and research” section and two in charge of “innovation and technology development.” To promote transdisciplinary research and capacity building at the regional scale, SACIDS created CoPs that encourage collaboration between institutions within or between member countries. Each CoP is led by two people, one from the medical side and one from the veterinary side, with the two leaders coming from different institutions and sometimes from different countries. The CoPs are organized around six research themes: (i) diseases of economic and food security importance, e.g., foot and mouth disease; (ii) emerging viral diseases, e.g., Ebola; (iii) bacterial zoonoses including food-borne diseases, e.g., tuberculosis; (iv) climate-dependent, vector-borne diseases, e.g., Rift Valley Fever; (v) bacterial rare diseases, e.g., plague; and (vi) cross-cutting OH issues, e.g., surveillance (which often require integrating different sciences). These communities of practice are composed of supervisors and mentors, postdocs, PhDs, and MSc students. Through research, everyone within a CoP should have the opportunity to interact and exchange with the other people of the CoP, to learn from them and to be a mentor for someone else.

**Figure 4:**
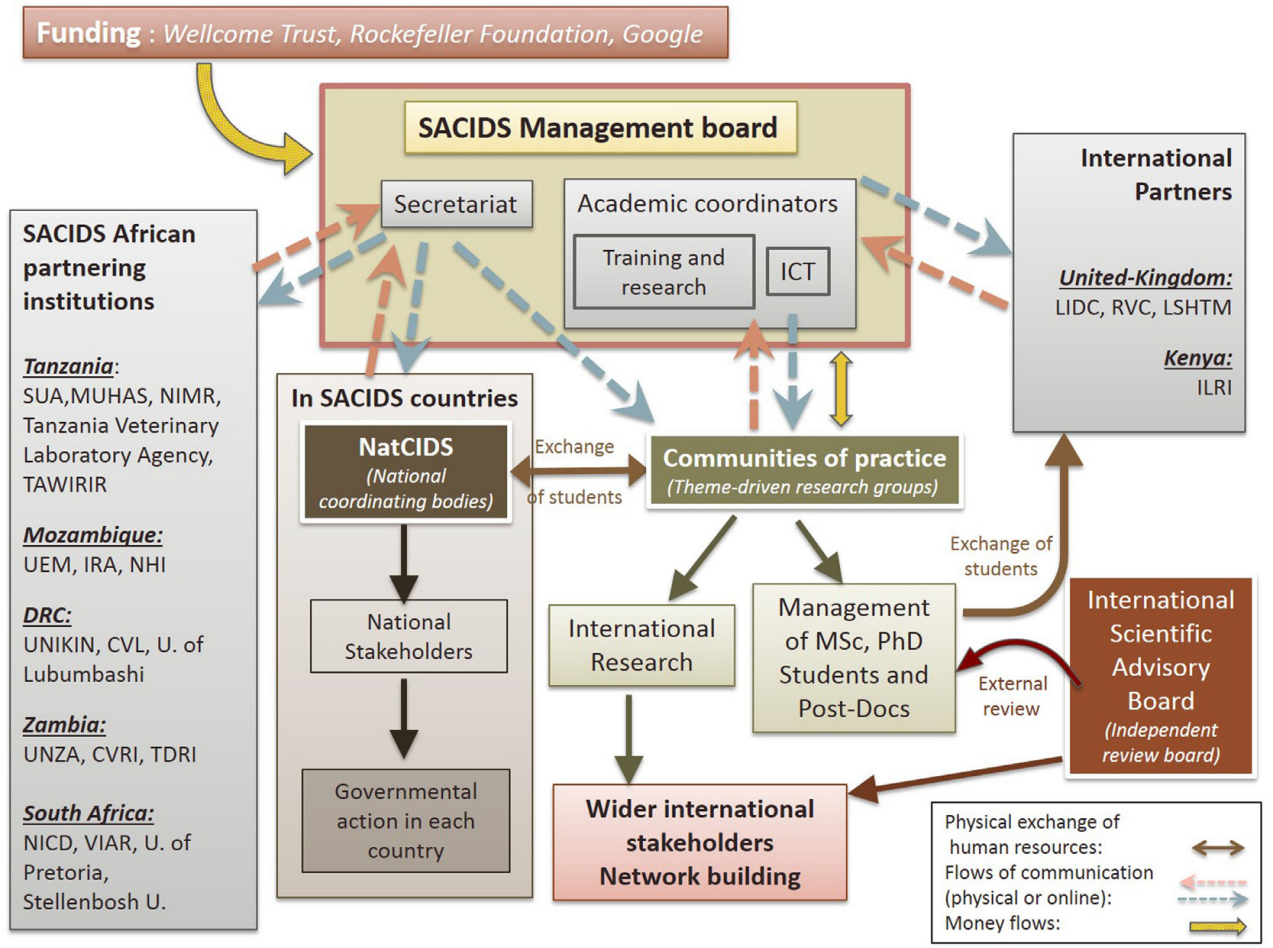
Interactions between Southern African Centre for Infectious Disease Surveillance (SACIDS)’ internal and external bodies. Abbreviations: SUA, Sokoine University of Agriculture; MUHAS, Muhimbili University of Health and Allied Sciences; NIMR, National Institute for Medical Research; TAWIRI, Tanzania Wildlife Research Institute; UEM, Eduardo Mondlane University; IRA, Institute of Agricultural Research; NHI, National Health Institute; UNIKIN, University of Kinshasa; CVL, Central Veterinary Laboratory; UNZA, University of Zambia; CVRI, Central Veterinary Research Institute; TDRI, Tropical Disease Research Institute; NICD, National Institute for Communicable Diseases; VIAR, Veterinary Institute of the Agricultural Research Council; LIDC, London International Development Centre; RVC, Royal Veterinary College; LSHTM, London School of Hygiene and Tropical Medicine; ILRI, International Livestock Research Institute; U., University.

At the national level, national coordination bodies are in place to ensure communication between the secretariat of SACIDS and the member institutions in the country (Figure 4). These structures, called the “NatCIDS,” are composed of at least two people, one with an animal health background and the other with a human health background. These NatCIDS are the relay to enable discussions between SACIDS and national stakeholders, which can facilitate the application of government decisions where necessary. They also have an important role in the communication between SACIDS countries, to facilitate the exchange of students for attending an MSc, PhD, or postdoc in another country.

Different funders provide funding at the level of the SACIDS secretariat for core functions for forum, coordination, communication, advocacy, and resource mobilization, among others. Each CoP is expected to mobilize their own resources and pay into the secretariat; in exchange they receive administration support from the SACIDS secretariat.

### Theory of Change and Outcomes of the Initiative

#### Theory of Change

**Figure 5:**
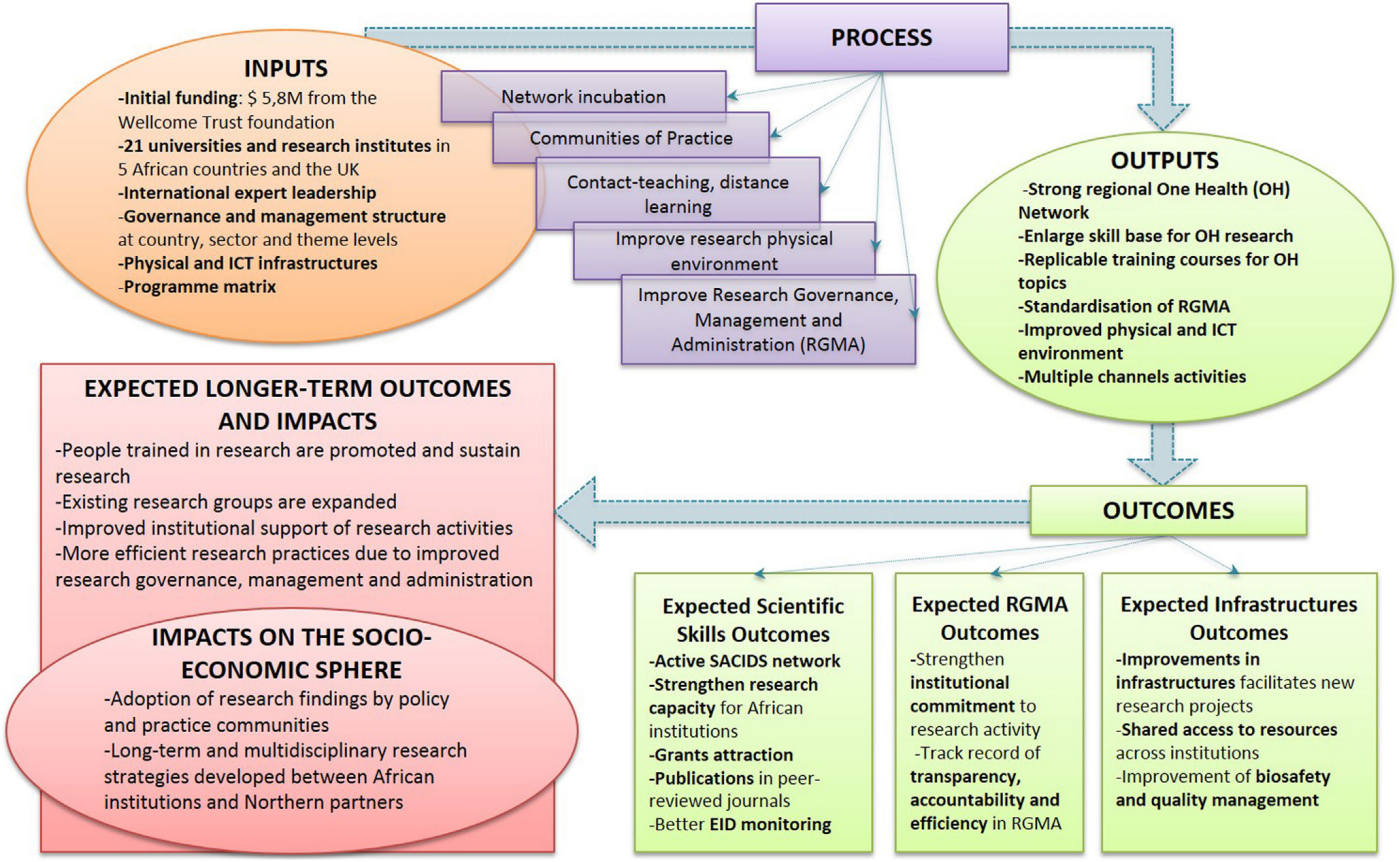
Theory of change of the Southern African Centre for Infectious Disease Surveillance.

In the first years of SACIDS, a model for a theory of change was elaborated. Building a theory of change is valuable for the implementation of long-term projects as it helps planning, implementing, and monitoring the required steps to reach the desired outcomes. SACIDS theory of change’s diagram, presented in Figure 5 [designed from Ref. (34)], shows the initial inputs received by the initiative in terms of funding, infrastructures, institutional expertise, management structure, and program strategy.

The process phase reveals the tools that SACIDS planned to use to create change in the African research and academic setting in which it evolves. This involved mainly network promotion, creation of communities of practice and teaching courses, and improvement of infrastructures and governance for research. The OH outputs that should arise from this process are core components to shape a compliant environment to conduce high-quality research at a regional level. From these, outputs should result in short-term expected outcomes, mainly structured in three categories: *Expected Scientific Skills* outcomes, *Expected Research Governance, Management, and Administration* outcomes, and *Expected Infrastructures* outcomes. In the long term, other outcomes and impacts on the socioeconomic sphere are expected to result from the SACIDS initiative. In the future, SACIDS initiative aims to lead to sustainable, well-funded, and well-managed African-led research, strong international scientific collaboration, as well as better consideration for research findings in policy adoption. However, these outcomes and impacts are for the moment only foreseen, and, at the time of the evaluation, SACIDS was still in the process of implementing changes at the institutional level and produce concrete outputs in the participating countries.

#### Measured or Estimated Outputs of SACIDS

At the time of this evaluation, SACIDS was still in early stages of program development; it has a long-term planning horizon and wants to continue building capacity for many years to come. Consequently, the timeframe to achieve outcomes and socioeconomic impacts as illustrated in Figure 5 do not fall within this first phase of SACIDS but remain open to achievements in the future. Nevertheless, SACIDS produced a broad range of outputs over the past 9 years. The key milestones put in place by SACIDS are presented for the main SACIDS’ objectives (Table 1), namely, enhancing BQM, enhancing ICT to support learning and disease surveillance systems, enhancing skills through taught and distance-learning programs, and enhancing skills through taught and distance-learning programs. The data used for developing Table 1 were extracted from yearly progress reports produced by SACIDS for the Wellcome Trust foundation.

**Table 1:** Southern African Centre for Infectious Disease Surveillance (SACIDS)’ outputs by objective and year.

	Enhance biosafety and quality management (BQM)	Enhance information and communication technologies to support learning	Enhance skills through taught and distance-learning programs	Enhance skills through research apprenticeships
2010		SACIDS website developed and running as www.sacids.org (accessed April 17, 2017)		

2011	Gap analysis of BQM at the participating university faculties/departments was conducted through quality auditing visits to participating institutions to establish their status, gaps, needs, and requirements for implementation of BQM systems	Linking of SACIDS Secretariat to the host institution’s financial system A website and a Facebook page were created to improve the consortium’s web presence	Launching MSc in One Health Molecular Biology (OHMB) took place with an intake of 8 SACIDS sponsored students (2 from Democratic Republic of Congo (DRC), 2 from Zambia, and 4 from Tanzania) First One Health (OH) Conference in Africa took place on the 14th and 15th July 2011 at the National Institute for Communicable Diseases (NICD)-NHLS, Johannesburg, South Africa	All Postdoctoral Research Fellows have been appointed to work on the following (disease) themes: Filoviruses, FMD, Plague, RVF, and TB

2012	Nomination of biosafety focal points for participating universities in the four countries (DRC, Mozambique, Tanzania, and Zambia)	Setup of videoconference equipment at Sokoine University of Agriculture (SUA), to facilitate online lectures Setup of high availability server environment for hosting e-learning software Deployment of a Google App implementation to assist students and SACIDS staff in document sharing and other online activities	Launching of the MSc One Health Analytical Epidemiology (OHAE) at UNZA was done. Twelve students were admitted by the University to the course OH curriculum workshop between the Royal Veterinary College, London School of Hygiene and Tropical Medicine (LSHTM) and SACIDS was held at the London International Development Centre (LIDC) on second April 2012 The First SACIDS Summer School 20–24th August 2012, SUA. This was the first One Health Summer School in Africa was jointly organized by SACIDS and LIDC	

2013		Two pilot e-learning systems were designed and deployed; custom made and open source (Moodle) Design, development, and deployment of a secretariat wide Intranet system as a tool to facilitate communication between and/or within the secretariat to improve data sharing capabilities and overall knowledge base	Second SACIDS Summer School Report of 20–24th August 2013, SUA	Research apprentices and their African and UK supervisors met on 14 April 2013 in Arusha for progress review, assessment of protocols and work plan development Six PhD students attended various transferable skill courses from 9th January to 8th February 2013 at the LSHTM

2014	Three mobile BSL-3 units for DRC (University of Kinshasa), Mozambique (Eduardo Mondlane University) and Tanzania (SUA) have been purchased to enhance biosafety for the diagnosis of and research on highly infectious diseases	Deployment of videoconference facility that assists students and research apprenticeships to virtual interaction with the supervisors and attend courses	Course modularization: modularization of MSc OHAE, modules for 7 courses have been written and finalized Third One Health Summer School August 25–30th 2014, SUA	The SACIDS community of practice (CoP) Leaders Meeting was held at the National Institute of Communicable Diseases of the National Health Laboratory Service (NICD-NHLS), Johannesburg, South Africa from 30th to 31st January 2014

2015			The modularization of OHMB Course at SUA has been completed	The molecular biology platform at SUA is in place Novel FMDV genotypes/topotypes have been identified Development of monoclonal antibody based lateral flow diagnostics for Ebola and Marburg diseases Discovery of two lineages of Peste des Petits Ruminants (PPR) Virus and of African swine fever co-circulating Discovery of novel topotype of *Mycobacteria bovis* in Serengeti ecosystem Finding of increasing AMR prevalence in southern and East Africa

2016		A new data collection tool (AfyaData) has been developed under a leveraged project funded project by Skoll Global Threat Fund, which is being used as disease surveillance tool. This tool is an improved version of previous tools and will also be used for data collection for various research activities	OHMB: a total of 36 students were enrolled for MSc OHMB since year 2010. Out of the 36 students, 3 were discontinued from their studies OHAE: a total of 26 students were enrolled for MSc OHAE since year 2011. Only two OHAE have not graduated	A total of 13 postdocs were recruited since 2010. Eight have completed their Postdoctoral Research and five are in their final stages of research work A total of 10 Res MSc were recruited. Seven have graduated

### Evaluation of the OHness of SACIDS

#### OH Thinking

**Figure 6:**
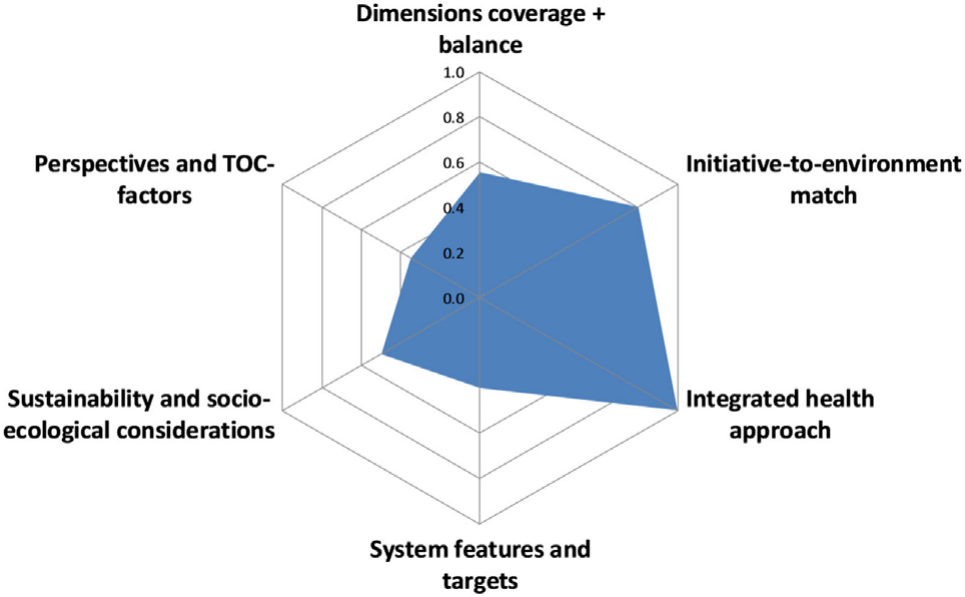
Assessment of One Health thinking in the Southern African Centre for Infectious Disease Surveillance.

The overall OH thinking score was 0.56 with a wide diversity of scores for the different aspects, ranging from 0.4 for system features and targets as well as perspectives and TOC factors, to 1 for its perceived integrated approach of health (Figure 6).

#### OH Planning

**Figure 7:**
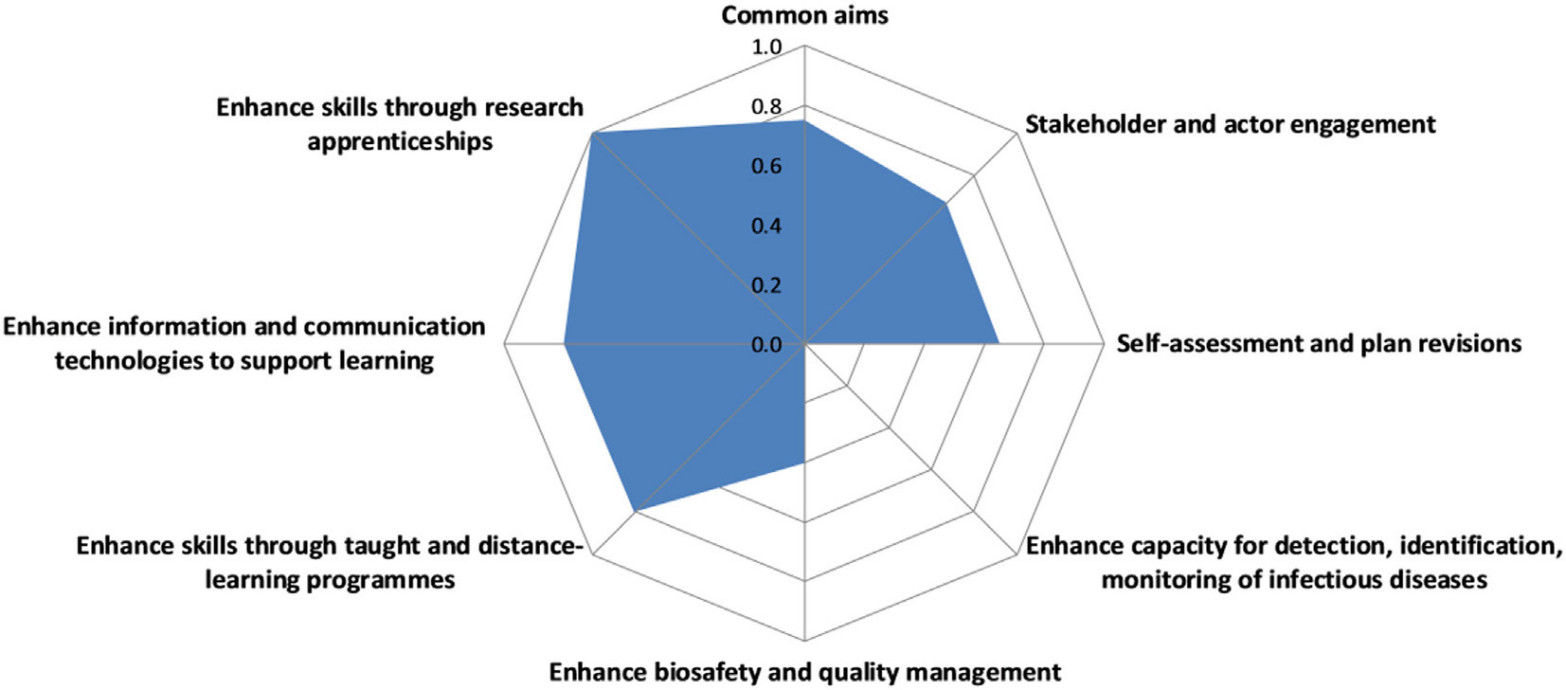
Assessment of One Health planning in the Southern African Centre for Infectious Disease Surveillance.

The overall OH planning score was 0.8 with some variation across scores for the different aspects, ranging from 0.4 for Objective 2, i.e., *enhance BQM*, to 1 for Objective 5, i.e., *enhance skills through research apprenticeships* (Figure 7). Objective 1 was not scored because of a lack of information for this objective.

#### OH Working

**Figure 8:**
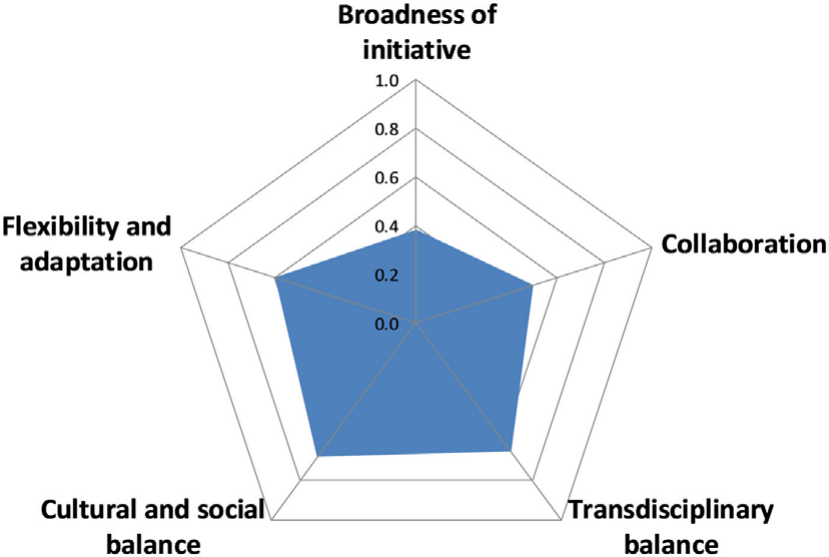
Assessment of One Health working in the Southern African Centre for Infectious Disease Surveillance.

The overall OH working score was 0.6 with some variation across scores for the different aspects, ranging from 0.4 for broadness of initiative and collaboration, respectively, to 0.7 for cultural and social balance as well as transdisciplinary balance (Figure 8).

#### Sharing Infrastructure

**Figure 9:**
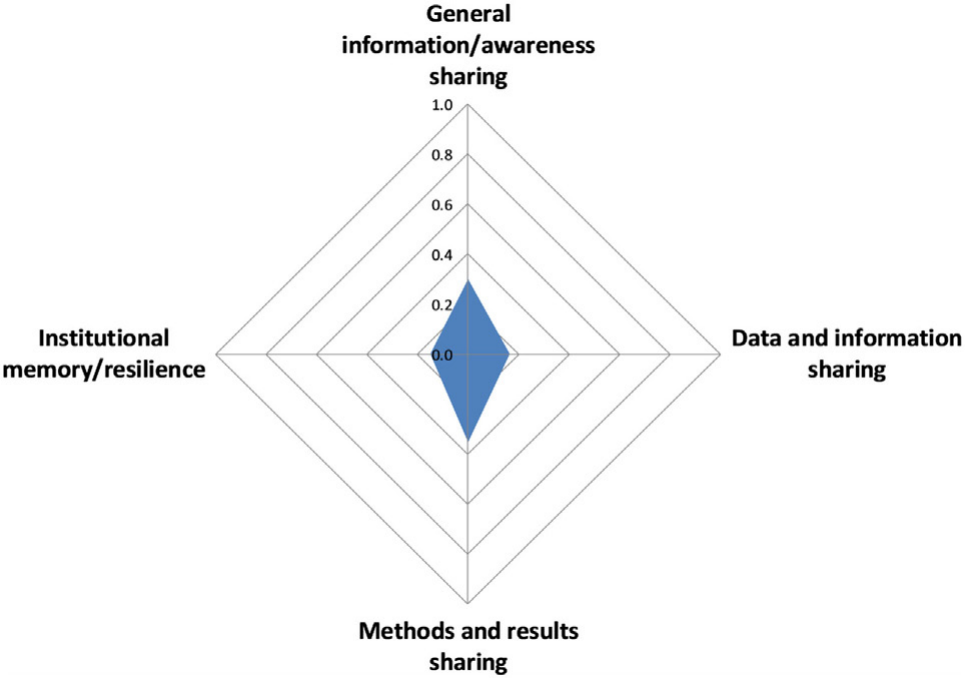
Assessment of information and data sharing in the Southern African Centre for Infectious Disease Surveillance.

The overall score for sharing information and data was 0.2 with little variation across scores for the different aspects, ranging from 0.2 for institutional memory/resilience to 0.4 for methods and results sharing (Figure 9).

#### Learning Infrastructure

**Figure 10:**
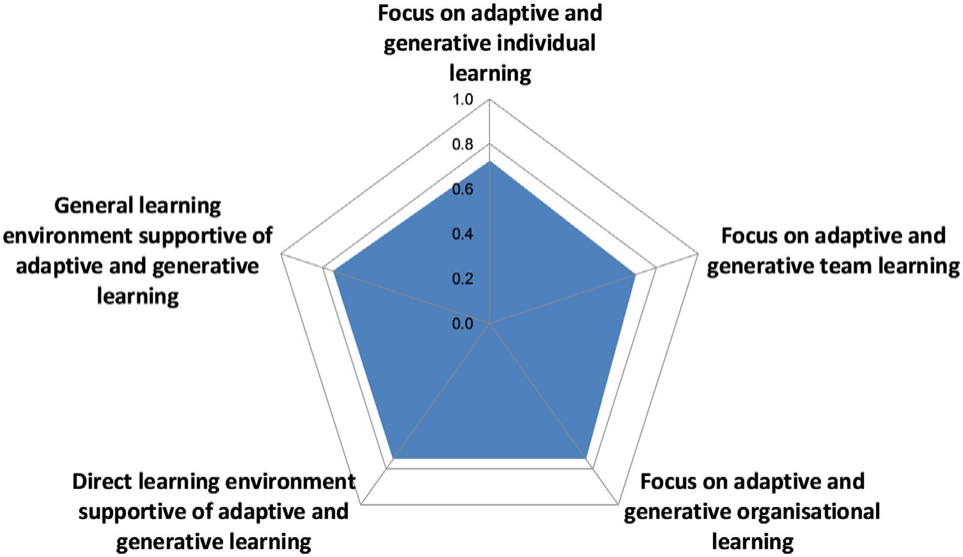
Assessment of learning infrastructure in the Southern African Centre for Infectious Disease Surveillance.

The overall OH learning score was 0.74 with very balanced scores of either 0.7 or 0.8 for all aspects considered (Figure 10).

#### Systemic Organization

**Figure 11:**
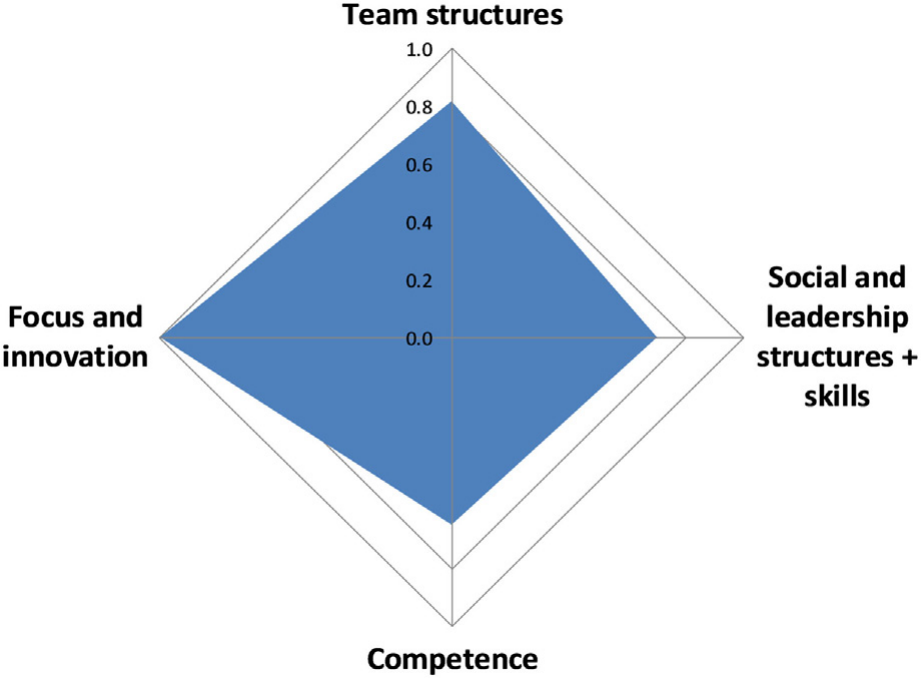
Assessment of systemic organization in the Southern African Centre for Infectious Disease Surveillance.

The overall score for the systemic organization in SACIDS was 0.8 with some variation across scores for the different aspects, ranging from 0.7 for social and leadership structures and skills as well as competence, to 1 for focus and innovation (Figure 11).

#### OH Index

**Figure 12:**
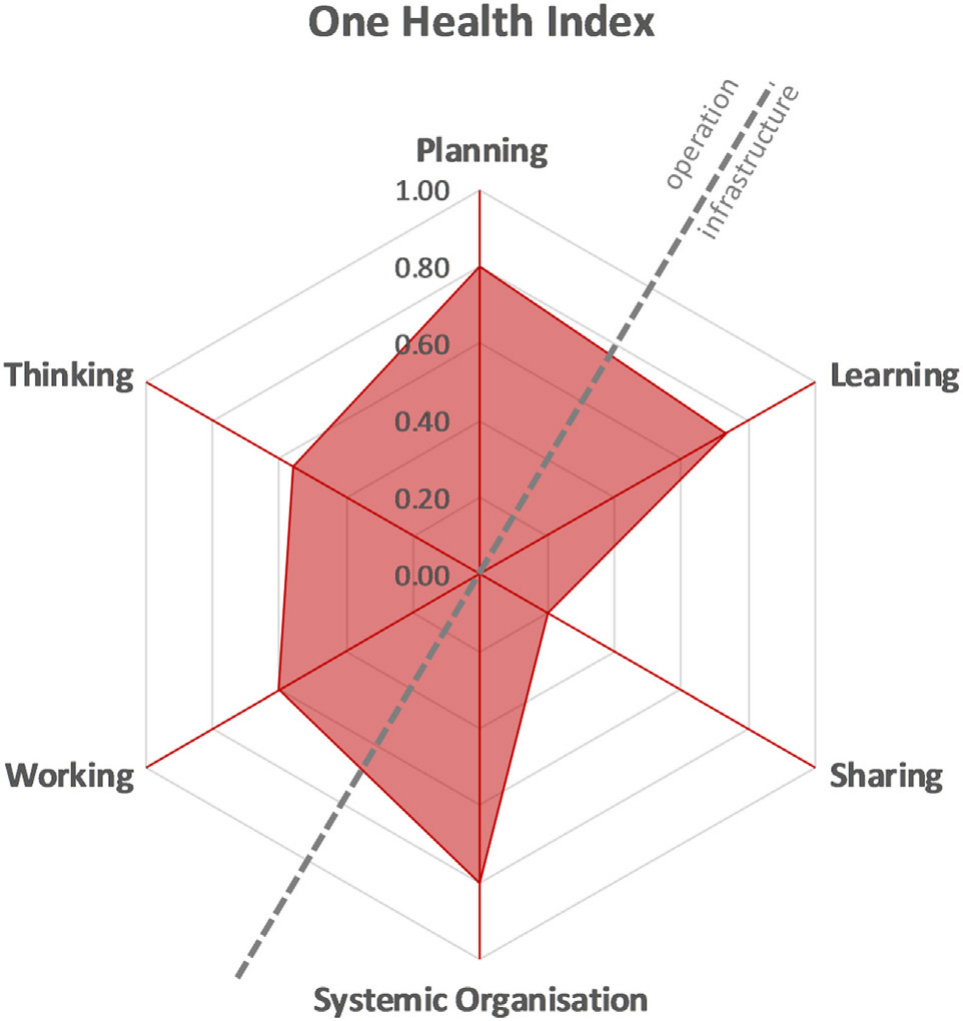
One Health Index of the Southern African Centre for Infectious Disease Surveillance.

Figure 12 depicts OH thinking, planning, and working opposed to learning infrastructures, sharing infrastructure, and systemic organization. The operational aspects on the left of the diagonal are opposed to the infrastructure on the right. Apart from a low sharing score, the two sides appear to be quite balanced. The OHI calculated was 0.359, and the OHR was 1.495.

### Pros and Cons in the Implementation of the Initiative

Southern African Centre for Infectious Disease Surveillance program participants stated that the main advantage of the program was its great innovative energy in a difficult landscape dominated by poor infrastructure and its ability to create awareness for OH and enthuse people for the concept. Another major positive observation was SACIDS ability to train a range of people in OH thinking and working who can be local enablers and have the skills to facilitate progress and development. They were described to be the people who understand that changes are necessary and can act as local champions to drive OH research, capacity building, and development considering the important pillars of OH.

The main shortcoming described by various SACIDS members was the imbalance across the countries involved in the South. Several people commented on the success of SACIDS in Tanzania and to some extent in Zambia and South Africa, but the DRC and Mozambique seemed to have been left behind in the process and struggled to make the same progress as other countries. Other points of criticisms raised related to a lack of data sharing, unequal allocation of resources, top-down management structures, and limited horizontal collaboration.

## Discussion

### Discussion of Evaluation Methodology

The evaluation and data collection process were well received by SACIDS participants. Both the SACIDS management and the secretariat were forthcoming in the engagement of respondents and supportive of the evaluation. All participants interviewed shared information, feedback, and opinions generously and appeared to answer questions honestly and trustfully. However, it was difficult to engage with more junior researchers in SACIDS, as they were busy with their projects or did not fulfill the inclusion criteria. The support of the SACIDS Executive Director was crucial in engaging SACIDS members and maximizing participation. Thus, the study has benefited from good representation of opinions across all SACIDS countries, apart from South Africa for which we were not able to arrange an interview within the available time frame.

Given that this was an external evaluation, the collation of documents, document review, preparation of questionnaires, and interviews were time-consuming activities. The complete question guide for the personal interviews was too long and too detailed to apply in full for all respondents. The interviewers therefore needed some flexibility in the way the questions were selected and asked, according to the specific circumstances (in particular background, knowledge, and expertise) of the interviewee. If respondents did not have enough knowledge on a specific topic, the interviewers moved on to the next question. Where respondents had ample in-depth insights and knowledge, the interviewers allowed more time for their answers. Consequently, there was an imbalance in the time spent on individual questions per interview, but the interviewers made an effort to achieve an overall balance across all interviewees.

The systematic approach developed by NEOH allowed insights into a wide range of aspects in a structured way, which may otherwise have been missed. Interviewees volunteered a broad range of information to open questions, often reflecting on what was asked before providing in-depth answers. Often, people gave lengthy and detailed answers demonstrating their own experience, self-reflection, and opinion of the program. The semi-structured interview format was thus perceived to be beneficial, as it allowed the gathering of data that could not have been predetermined.

The evaluation was conducted primarily by the first and last authors, both of whom were external to SACIDS, although one assessor had collaborated with a SACIDS project previously. Consequently, there was no conflict of interest, and the assessors were able to conduct the study in an unbiased manner. The information shared was often based on personal experience and subjective impressions, but the approach to include a broad range of representatives from across SACIDS allowed to gain a comprehensive picture and a good understanding of the global functioning and processes of SACIDS. However, an even more detailed understanding of the theory of change, the SACIDS operations and some of the individual concerns reported could have been gained by using participatory approaches with representatives from all SACIDS institutions. Due to time and budgetary constraints, this was not feasible but would have provided further information on commonalities and differences in the vision and the implementation of SACIDS. The collection of such data could be considered during one of the SACIDS annual meetings in the future.

Finally, it was observed that the evaluation at the program level may not have captured some of the more integrated and transdisciplinary endeavors of single research projects. This indicates a methodological shortcoming of the NEOH framework for the evaluation of a large program aimed at capacity building. One of the respondents mentioned that there are fewer divisions between the different disciplines in Africa driven by “living in a disease rich environment, the economics, the malnutrition, the poverty and tough life in general” (respondent observation). Thus, researchers often have advanced skills working within communities, know alternatives to conventional medicine, and are more flexible to adapt to changing circumstances. These issues were poorly captured during the data collection for this evaluation.

Because of resource limitations, this study only listed outputs in relation to the theory of change but did not investigate outcomes achieved and their classification into disciplinary, interdisciplinary, or OH outcomes. At the level of the output, the OH concept is difficult to distil; interdisciplinarity is more likely to be noticeable at the level of the outcomes. In future research, efforts should be placed on the assessment of the outcomes achieved and the impact they may provoke.

### Discussion of Results

This part refers to the OHness scores reported in the results section and the associated information and justification in Table S3 in Supplementary Material. SACIDS was a very ambitious and visionary program driven by the need to fill existing gaps in surveillance capacity of endemic, exotic, and emerging diseases in Southern African countries. Coordinating a program across different countries, sectors, and disciplines with different cultures and context comes with many challenges. The program planning was based on a needs assessment of surveillance capacity, and the acknowledgment that an integrated approach to health was called for. From the outset, SACIDS aimed to be an African-led initiative that would produce capacity to address issues of societal, environmental, economic, and health concerns through an interdisciplinary, OH approach. The program was complimented by many respondents for its ambition, the changes achieved and the effectiveness in bringing together disciplines and sectors in a shared program.

This evaluation was conducted at the level of the SACIDS program and not the individual projects. Consequently, the results do not report on the finer detail related to diseases, associated technical capacities, and field work that would be found at the level of research projects within the CoPs.

For most OHness dimensions, apart from information and data sharing infrastructure, SACIDS scored between 0.6 and 0.8.

With regards to OH working, SACIDS presented an effective and productive management structure that clearly addressed issues in the context it operates tackling underlying disconnect and silos among different sectors. Its setup spans different countries, sectors, and various types of institutions with the aim to build capacity in an integrated manner. An external feedback mechanism from the International Review Board promotes reflection and action for change when needed. SACIDS actors have proven to be flexible in their working and are able to react to changing circumstances, as observed by one interviewee: *The director is able to change his thinking and his staff seem able to do the same*. Despite the flexibility observed, there were some shortcomings in that the working was perceived to be rather hierarchical with little engagement of junior people in decision-making and no perceptible involvement of grass-root organization or communities in the coordination and management of the program.

While the teams were described to work very well within the CoPs, they existed as clear units within institutional teams with little interaction with other disciplines, although increasing interaction between the human and veterinary teams was reported to be developing. Increased interaction could be nurtured by having more dedicated staff members that would actively link members and encourage exchange and team building in an integrated way. Currently, there is only one person acting as the main coordinator between SACIDS and consortium partners. This role could also extend to linking more effectively with Northern partners and promoting a professional collaboration based on equality. The current partnership was perceived by some to be unbalanced with behavior of superiority instead of equality. Nonetheless, the model of joint supervision with input from Northern partners was highly appreciated and perceived to be effective and beneficial.

The Southern African Centre for Infectious Disease Surveillance earned praise for its recruitment process of high academic quality postgraduate students. It was suggested to use a similar recruitment process within other SACIDS functions with the aim to increase the breadth of people from different countries, cultures, and expertise. In addition, this would enhance the transdisciplinary skills in the program (or as one respondent put it: *allow SACIDS to have a cocktail of ideas and understandings*) and reduce the bias toward selection from Tanzania. This could be achieved by promoting self-reflection and feedback in SACIDS. The current structures were found to be well established and working well with regards the implementation of the program and the technical considerations, but there seemed to be little input into management and organizational structures across all SACIDS members. Because transdisciplinarity is based on broad engagement of actors and stakeholders, such a move could help to create more ownership and networking. The respondents made several useful recommendations during the interviews on how the SACIDS organization could be improved. They included expanding of SACIDS to allow new institutions and people to join the program; a more communicative, engaging, and disseminative secretariat that would actively link all partners; a clear definition of roles and responsibilities for everybody involved; and promotion of a more participatory approach that would help establish consensus from a broad range of participants including people on the ground (as mentioned by one respondent *We need to get a degree of democracy within SACIDS. The ownership has to go to the people*). These examples show that there are several suggestions for change available in the consortium, and the program may benefit from harvesting the creative potential in a participatory way.

The lowest score overall was recorded for information and data sharing (score of 0.2). Several concerns were voiced relating to this issue. Although one of SACIDS’ primary aims was to create a trans-national network to enable transdisciplinary collaboration between actors engaged in the control of infectious diseases, there is a lack of resources attributed to information and data sharing and the creation of institutional memory. Each CoP team is in charge of data quality, with combined responsibility from the supervisor and the team. To date, there is no student induction program, which means that the supervisor’s research experience is relied on to ensure data quality, rather than a systematic approach, which can lead to variability in data quality between students and between projects. While there are some structured internal sharing mechanisms and established ways of communication between CoPs, SACIDS lacks a platform where data could be shared and made available among project partners in a standardized format that would reduce risks of duplication. In addition, there are no formal institutional arrangements that would support data and information sharing in a systematic way. Consequently, there is real risk that institutional memory may be lost if knowledgeable SACIDS members move to new jobs without passing on their knowledge to others. Currently, the program only has a repository for results (e.g., theses, publications) but could benefit from a common database or documentation that stipulates how data must be checked, prepared, and presented for sharing purposes. While the establishment of such a database can be resource and time consuming, the establishment of a shared archive may be more achievable. With such a resource, all SACIDS partners would have access to the outputs and would not need to go through the secretariat if they wanted to access certain information. A “read-only” database would minimize the risk of mistakenly deleting items from the repository or changing them. A benefit of such a database could be to make educational materials more widely available so that partners could use them for teaching and training purposes. Some respondents observed that SACIDS had contributed greatly to sharing of information between individuals or groups by enabling better cooperation and collaboration between them, but that there was large individual variability and many people or institutions were still reluctant to share data with others. This may be an indication of the tensions that may exist between individual interests for (academic) progress and a need to share information and data for the purpose of a common good, which should be an underlying principle of a program like SACIDS. Given the importance of data and information sharing in transdisciplinary programs and the efficiency loss associated with insufficient institutional memory, SACIDS may want to consider making an investment into formalizing and promoting such processes.

At the regional management level, certain processes were reportedly sluggish because of delays in implementation, sub-standard logistical processes, and unclear lines of command. Consequently, this affected the performance of researchers and projects, being unable to proceed due to delays in accessing the required materials. Previous evaluations of SACIDS already reported challenges related to the distribution of resources (12). Coupled with the perception that there is a bias in funds, staffing, and activity toward Tanzania, some participants suggested decentralizing some of the secretariat’s activities and funding, to allow some national level management and facilitate resource allocation at country level. Because SACIDS only pays the secretariat and the Executive Director, some of the other SACIDS participants with in-kind contributions feel that they are undervalued for the time invested. Consequently, there was a call to generate more funding and create more paid positions across all SACIDS countries in a transparent way with the aim to institutionalize the program and reduce bureaucratic burden on SACIDS members.

The unequal success in the implementation of postgraduate programs across SACIDS countries can partly be explained by different institutional capacities at the start of the program. Unlike Zambia and Tanzania, DRC and Mozambique did not have any PhD programs in place before the start of SACIDS, and the implementation of such courses required more time and effort than in the other countries. In addition, Zambia and Tanzania institutions benefit from being designated as “African Centres of Excellence” by the World Bank, which reflects the high quality of research and educational programs in place in these countries. SACIDS seems aware of the necessity to address the need for better capacity building in DRC and Mozambique and is prioritizing this issue as a major objective for the next phase of the program.

Because this evaluation was conducted at a stage, where the program development had mainly focused on its establishment (e.g., network, training and capacity building, development of infrastructure and processes), there is currently a disconnect between the rationale and motivation for SACIDS and actual outcomes and impact. Consequently, there is no assessment of improvements in attributes that define infectious disease surveillance, such as performance or functional attributes of surveillance or their OH integration. To facilitate future program success and achievement of outcomes and impact, it is recommended that SACIDS develops measurable indicators in line with their theory of change and implements relevant data collection and evaluation activities. Such information will not only be important for the management of SACIDS but also enhance credibility among (future) funders.

Across the board, people perceived SACIDS to be a very positive and effective African-led initiative that helps bring together the disciplines of OH and allows important issues in the region to be addressed and to bridge funding gaps. Nowadays, there is a common understanding that we need both a quantitative and qualitative approach to understand, prevent, and control infectious diseases. This cannot be done with classical veterinary interventions alone, such as vaccination or other technical measures, but it is important to consider other perspectives as well in a systems approach. The focus of SACIDS has been primarily on technical capacity building and the shortcomings relating to environmental disciplines and the social sciences, which it aims to address, are acknowledged and will be addressed in the next phase of the program. SACIDS’ vision and mission are important endeavors for capacity building in the region and its approach has the potential to promote progress and development. However, this evaluation has identified several issues in management, delivery, and OH integration that the program may want to address to promote success in the long term and realize the outcomes and impact envisaged in an efficient and effective manner. It is recommend that SACIDS continues to look at how the technical progress can be embedded in social aspects, local communities’ practices and behaviors. Consequently, it may be important to look at other measures that may lead to changes in infrastructure, such as sewage and water systems, land management policies, and education. A prioritization approach based on participatory engagement with a wider representation of sectors and disciplines from all strata of society could inform a process of discussing strategic directions and grant applications for the future.

## Ethics Statement

This study was carried out in accordance with the recommendations of the ethics committee of the Royal Veterinary College with written or oral informed consent from all subjects. All subjects gave informed consent in accordance with the Declaration of Helsinki. Ethical approval for the interviews and the online survey was sought from the Royal Veterinary College and was granted by its Social Sciences Research Ethical Review Board, number URN SR2017-1002.

## Author Contributions

MH and BH conceptualized the study with guidance from SR and SS. MH, KQ and BH were responsible for data collection with EK facilitating access to data, reports, and networks. MH and BH had primary responsibility for data analysis and manuscript writing with all coauthors giving feedback and input.

## Conflict of Interest Statement

The authors declare that the research was conducted in the absence of any commercial or financial relationships that could be construed as a potential conflict of interest. The handling editor and author SR declared their involvement as coeditors in the Research Topic and confirm the absence of any other collaboration.

## Footnotes

^1^The SADC comprises 15 countries: Angola, Botswana, DRC, Lesotho, Madagascar, Malawi, Mauritius, Mozambique, Namibia, Seychelles, South Africa, Swaziland, United Republic of Tanzania, Zambia, and Zimbabwe.

## Abbreviations

CoP, community of practice; ICT, information and communication technologies; NEOH, Network for Evaluation of One Health; OH, One Health; OHAE, One Health Analytical Epidemiology; OHMB, One Health Molecular Biology; RGMA, Research Governance, Management, and Administration; SADC, Southern African Development Community; SACIDS, Southern African Centre for Infectious Disease Surveillance.
