# Supplementary material for: A One Health Evaluation of the Southern African Centre for Infectious Disease Surveillance
Source: Front Vet Sci. 2018 Mar 16;5:33. doi: 10.3389/fvets.2018.00033 (PMC5864892; doi:10.3389/fvets.2018.00033)
Supplement: Supplementary file 1 [file Table_1.PDF]

Question guide for personal interviews for the One Health evaluation of SACIDS (OH surveillance capacity building)

Prepared by Barbara Haesler based on the tools of the Network for Evaluation of One Health (NEOH, http://neoh.onehealthglobal.net/), April 2017

Interview date: *Name of interviewer:*

I. Introductions

II. Consent:

Dear Sir/Madam,

Good morning/afternoon and thank you for your interest in our study. My name is ………………………. and I am working with a team from the Royal Veterinary College in London to investigate the One-Health initiatives put in place by SACIDS over the past 8 years. In this interview, I will ask you questions in relation to the SACIDS objective on capacity building for infectious disease surveillance in a One Health framework. This study aims to evaluate and measure the "One-Health-ness" of SACIDS capacity building in the surveillance of infectious diseases, so we will ask you questions regarding SACIDS internal functioning, as well as ask about your view on ways of working within SACIDS activities.

Assessing the efficiency of these One-Health initiatives can help SACIDS be aware of its strengths and weaknesses and gain efficacy in One-Health capacity in future years. Evaluating One-Health initiatives is something new that will need standardised methods in the future. Our study also aims to test and improve the design of protocols developed specifically to evaluate One-Health projects like SACIDS.

It is entirely your choice to decide whether or not you wish to participate in our study. If you decide to take part, you can change your mind later on and withdraw from the research study. The decision to join or not to talk with us today will not cause you to lose any benefits. If you do not feel comfortable answering any of the questions just let us know and we will move on to the next question. You are also free to withdraw from the interview at any moment without giving any explanation.

The information you give us is completely confidential, and we will not associate your name with anything you say. No reference to specific people will be made in any written or oral presentations and your responses will only be used for research purposes.

We would very much appreciate your help and collaboration in this project. Your participation is important to the success of the study but it is entirely voluntary and unwillingness to participate will not preclude you from access to the results of the research. We believe the results of this project would be useful for you and we would be happy to communicate the results when the study is completed.

Please feel free to ask us if you have any questions regarding this questionnaire or regarding the project in general.

Do you agree to take part in this study: □ Yes □ No

III. Activity 1: Overview, context, ToC, stakeholder mapping

i. What is the context in which the initiative is taking place, what leads to the implementation of a One Health initiative?

ii. What is the objective of the initiative to evaluate?

iii. What is the rationale of the One Health initiative?

iv. Which dimensions and scales are concerned by motive of the initiative?

1. Writing a **narrative** to explain the logic of the initiative.
2. Identifying basic assumptions about the **context.**
3. Identifying long-term goals
4. Backwards mapping and connecting the **preconditions or requirements** necessary to achieve that goal and explaining why these preconditions are necessary and sufficient.
5. Identifying the **interventions** that the initiative will perform to create the desired change.
6. Identifying and/or developing **indicators to measure outcomes** to assess the performance of the initiative.

Questions: Tell me about the history of SACIDS in Tanzania/Zambia in relation to the first objective. Why did you feel that something like SACIDS was needed? What did you hope to achieve? What was the situation then? Did you make certain assumptions? What is the situation now? Why did you establish SACIDS? What is its rationale? What will be the long-term goals and changes that you are hoping to achieve/have achieved already? Who was involved at the start to create capacity for OH surveillance for interdisciplinarity? Who joined/left over time? Were there stakeholders that you tried to engage, but it did not work?

IV. Activity 2: Evaluation of data and information sharing in One Health

Stakeholder definition according to the NEOH handbook glossary: any individual, group or organisation who may affect, be affected by, or perceive themselves to be affected by, a decision or activity.

Actor definition according to the NEOH handbook glossary: any individual, group or organisation who acts, or takes part in the One Health initiative and its context. In contrast to "stakeholder", an actor is not only affected, but takes part in the One Health system

	Evaluation elements	Short element description	Relevant in this initiative?	Describe assessment points	Qualitative assessment (describe)
	(Name of the element)	(Description and references, if needed)	(Used for normalisation of overall score. E.g. if not using data, then variable description is not necessary and would therefore get 0, but if data used then variable description needed = 1)	(E.g. almost all variables in the databases used in the initiative are described carefully in English, ensuring a common understanding of what they mean or were derived from raw data)	(E.g. non-existent, very low level, poor, good, high level, excellent depending on what is being evaluated)

1.	Stakeholder* identification process	Has a process to identify and involve all essential stakeholders (including governmental, academia, industry, NGOs, general population, etc.) been described and followed in the initiative?	Q: Can you describe the process used to identify the essential stakeholders and actors for capacity building?		
2.	Stakeholder involvement	Have essential stakeholders (including governmental, academia, industry, NGOs, general population, etc.) been involved at an appropriate level throughout the duration of the initiative?	Q: Did stakeholders and actors engage at the necessary level in SACIDS? Were there stakeholders that did not engage sufficiently?		
3.	Actor** identification process	Has a process to identify and involve all essential actors (including governmental, industry, health units, professionals, technicians, etc.) been described and followed in the initiative?	Q asked above to avoid repetition of the question		
4.	Actor roles and commitment	Do all essential actors (including governmental, industry, health units, professionals, technicians, etc.) have the opportunity to fulfil their roles and are they committed appropriately throughout the duration of the initiative?	Q: How committed are these actors? Do they have the opportunity to fulfil their roles? Are there differences among actors?		
5.	Internal information sharing mechanism	Does the initiative have appropriate mechanisms in place to facilitate sharing of information within the initiative? (E.g. newsletters, workshops, reports available to all, results getting published, online information sharing platform……)	Q: how do you share information within SACIDS?		
6.	External information sharing mechanism	Does the initiative have appropriate mechanisms in place to facilitate sharing of information outside the initiative? (E.g. newsletters, workshops, reports available to all, results getting published, online information sharing platform……)	Q: how do you share information outside SACIDS in Tanzania (e.g. to stakeholders)?		
7.	Sharing resources	Have resources been allocated to ensure necessary data and information sharing?	Q: Have you allocated resources specifically to the sharing of data and information?		
8.	Data sharing agreement	Have appropriate (e.g. formal/written/signed) agreements been made concerning data sharing in the initiative?	Q: Do you have any formal agreements in place? What are they?		
9.	Data quality	Are mechanisms/ procedures in place to ensure data quality, e.g. data completeness, error-checking and correction of errors, variable descriptions, description of aggregations/ calculations, documentation available.	Q: What mechanisms are in place to ensure data quality? Are they the same across all projects/actors/institutions?		

10.	Data storage	Are mechanisms/ procedures in place to ensure safe and appropriate data storage? (e.g. type of software, server, backup)	Q: What mechanisms are in place to ensure data storage?		
11.	Data accessibility	Are mechanisms/ procedures in place to ensure safe and appropriate data accessibility to facilitate sharing? (e.g. is extraction of data feasible without access to experts, or are experts readily available for extraction of data, is the process of data extraction bureaucratic/ cumbersome/overly time-consuming?)	Q: How accessible are the data?		
12.	Data sharing	How well are data being shared between people within the initiative? (e.g. compartmentalised (score 0-33), shared between few groups (34-66), fully shared between all in the initiative (score 67-100).	Q: How are data, methods and results being shared? Between whom?		
13.	Method sharing	How well are methods shared between people within the initiative? (e.g. compartmentalised (score 0-33), shared between few groups (34-66), fully shared between all in the initiative (score 67-100).	Included above		
14.	Results sharing	How are results shared between people within the initiative? (e.g. compartmentalised (score 0-33), shared between few groups (34-66), fully shared between all in the initiative (score 67-100).	Included above		
15.	Institutional memory	How well does the initiative include the creation of potential institutional knowledge reservoirs for data, methods and/or results over time?	Q: Are there mechanisms in place to promote institutional memory? What are they?		
16.	Resilience to change	Are mechanisms/procedures in place to safe-guard data and information access in case of system change, e.g. change of IT-system, data ownership, institutional organisations.	Q: How safe are the data you are producing when it comes to changes in staff, data ownership, institutional organisation, etc?		
17.	Use of information in learning	To which extend are data and information produced in the initiative used for learning/education activities?	Q: In what way do you use the data produced for learning? On a scale from 1 to 100?		

V. Activity 3: Assessment of OH planning

The elements considered in One Health planning derive from One Health thinking. To capture them, complete Table 3 on tasks, responsibilities, authority and means.

Describe tasks first. Use the stakeholder(s), dimension(s) and scale(s) identified in Section 0 to characterise the task. Then describe the responsibilities of the stakeholder(s) involved. Thirdly, describe the authority the stakeholders have in the different affected dimension(s) and at the different scale(s). Finally, record the means in terms of resources, staff and financing that are available to each stakeholder for the specific task. Finally, evaluate the match of responsibilities, authority and means, where 1 is a perfect match, 0.5 is a partial match (either too little or too much), and 0 is no match at all. To evaluate the overall planning, compute the mean match over all stakeholders and tasks.

Table 1. Tasks, responsibilities, authority and means in One Health planning for One Health surveillance capacity building

	Task	Stakeholder	Responsibility	Authority	Means	Match
		Q; who are the stakeholders? Describe them.	What responsibilities did the different SACIDS partners have?	What are their levels of authority?	How many people and other resources (money, infrastructure, etc) – enough to facilitate the establishment of this network? Shortcomings?	
18.	Establish and sustain network with connected partners					
19.	Enhance the capacity of institutions for the detection, identification and monitoring of infectious diseases of both humans and animals, in a 'one medicine' framework.					
20.	Enhance biosafety and quality management.					
21.	Enhance skills through taught and distance-learning programmes (masters and CPD courses).					
22.	Enhance ICT to support learning.					
23.	Enhance skills through research apprenticeships (PhDs and post docs)					

VI. Activity 4: Assessment of SACIDS transdisciplinarity

Enhance the capacity of institutions for the detection, identification and monitoring of infectious diseases of both humans and animals, in a 'one medicine' framework.

	**Assessment of OH Working**
24.	What was/is the problem with the former/existing capacity?
25.	To which aspects of OH, i.e. Human, animals and environment is this initiative relevant?
26.	Is transdisciplinarity (TD) required to solve this problem? What are the benefits of using TD rather than a conventional (*disciplinary*) approach? (*TD= a research strategy that crosses many disciplinary boundaries and involves stakeholders to create a holistic approach)*
27.	Which stakeholders and/or actors are involved? State the groups, individuals and dimensions that are concerned by the problem (concept/mind mapping). (*Stakeholder = any individual, group or organization who may affect, be affected by, or perceive themselves to be affected by, a decision or activity of the OH system; Actor = any individual, group or organization who acts, or takes part in the OH system)*
28.	How has the system evolved in terms of: stakeholders, actors, restrictions, consequences, pathways and boundaries?
	**Assessing the broadness of the Initiative**
29.	How diverse are the disciplines, methods, scales of analysis and/or social actors involved? (*i.e. enumerate all disciplines, methods, dimensions and scales of analysis considered, as well as the social actors involved.)*
30.	Is the non-scientific community involved? If yes, how? And if no, why?
	**Assessing Integration**
31.	To what extent is the project/initiative inter-sectorial? Which sectors are involved?
32.	To what extent do the different disciplines work together?<br>(i) Are meetings with all disciplines (face-to-face or virtual) held frequently?<br>(ii) Are aims and objectives shared and clear to all?<br>(iii) Is there joint decision-making?
33.	Are there imbalances of power (i.e. academic or disciplinary dominance) within the group? Is there a risk of bias in the process?<br>(i) Across disciplines<br>(ii) Across sectors<br>(iii) Across ethnicities<br>(iv) Across social classes<br>(v) Across gender<br>(vi) Other social difference (please specify)
34.	What are some of the contexts that have/may inhibit success of the initiative? e.g. cultural / religious issues
35.	What is the spatial proximity among disciplines' offices? (*probes for readiness for collaboration.)*
36.	Are there face-to-face interactions? If yes, how frequent are these interactions? (*probes for readiness for collaboration)*
37.	How innovative and how suitable is the combination of disciplines and fields of expertise for the specific purpose?<br>How do they select whom to involve in what? (*Assess originality and suitability of the combination of disciplines and fields of expertise for the specific purpose)*
38.	How balanced is the weaving of disciplines or fields of expertise? (*The question assumes that an integration that balances disciplines or fields of expertise is a sign of high inter- and transdisciplinary quality.)*
39.	Is a common One Health objective formulated that covers all the disciplines? (*The question checks for One Health objectives in transdisciplinarity and for knowledge integration)*
40.	To what degree can an OH objective serve as a basis for knowledge integration? (*assumes that an integration that balances disciplines or fields of expertise is a sign of high inter- and transdisciplinary quality.)*

	**Assessing Reflection and learning**
41.	Is the approach to self-reflection, learning and adaptation at individual, team and/or institutional level? (Q*uestion assumes that planned stages of self-reflection and learning and the possibility to adapt the case study based on this is a sign of high inter- and transdisciplinary quality.)*
42.	How likely is reflection going to feed back into corrective action within the case study? (*The question asks the reviewer to assess whether the case study will connect reflection and action.)*
43.	How flexible is the project design and timeline to respond to internal or external changes at short-term? (*The question checks for feasibility of project design and timeline in the short-term.)*
44.	How flexible is the project design and timeline to respond to internal or external changes at mid-term? (*The question checks for feasibility of project design and timeline in the medium term*.)
45.	How flexible is the project design and timeline to respond to internal or external changes at long-term? (*The question checks for feasibility of project design and timeline in the long-term.)*
	**Assessing efficiency and effectiveness of the initiative**
46.	How elaborate is the initiative and its specific contribution to solving the problem? (*The goal is to determine if there are problems they feel they haven't addressed.)*
47.	To what extent does the initiative establish mechanisms for capacity building beyond any previous (attempted) initiatives? (*Assess whether the case study will support future problem solving)*
	**Assessing Management, social and leadership skills**
48.	What are the management structures involved in this initiative? Assess their roles, capacity, challenges, disciplinary composition and fields of expertise etc. (*An elaborate management structure is a sign of high inter- and transdisciplinary quality)*
49.	What is the type of leadership is demonstrated in this initiative?<br>*a.* Small and collated *(Single leader, central leader, informal connections, face-to-face processes, teambuilding, leader needs process skills*<br>*b.* Large and dispersed *(Multiple leaders/champions, leaders in brokerage positions, coordination needed among leaders, leaders as translators and conflict handlers)*
50.	How would you characterize the leadership in the initiative in regard to task-orientation, relationship-orientation and change-orientation
51.	Does the initiative demonstrate open mindedness? (*The question assumes that if the case study shows core values of inter- and transdisciplinary ethics, this is a sign of high inter- and transdisciplinary quality.)*
52.	Does the initiative demonstrate self-reflection? (*The question assumes that if the case study shows core values of inter- and transdisciplinary ethics, this is a sign of high inter- and transdisciplinary quality.)*
53.	Does the initiative demonstrate changing hierarchies? (*The question assumes that if the case study shows core values of inter- and transdisciplinary ethics, this is a sign of high inter- and transdisciplinary quality.)*
54.	Does the initiative demonstrate ability to bear and manage tensions? *The question assumes that if the case study shows core values of inter- and transdisciplinary ethics, this is a sign of high inter- and transdisciplinary quality.*
	**Assessing Team structure (well structured vs pseudo team)**
55.	Is teamwork part of this initiative? (*The question focuses on whether the case study is based on teamwork. The question assumes that if the case study shows teamwork across disciplines, this is a sign of high inter- and transdisciplinary quality.)* Describe the measures taken to encourage team work? (*The question checks for practical measures that foster teamwork across disciplines)*
56.	How many teams do you work with? How are inter-team relations fostered? (*Probe for challenges experienced (what has worked and what has not). The question checks on the number of teams involved in the initiative: one, two, three or more teams related to this initiative? The question assumes that the more complex the case study, the more teams are present.)*
57.	If more teams than one are mentioned, how good are the inter-team relations? (*The question probes for how well different teams work together for the overall aim of the solving the problem. The question assumes that if the case study shows good inter-team relations, this is a sign of high inter- and transdisciplinary quality.)*
58.	Does the team have clear objectives? How are the objectives set? (*Probe for challenges experienced when setting objectives (what has worked and what has not) The question determines whether the team/s are well structured or not?*)
59.	Do team members work closely together to achieve the team's objectives? (*Probe for challenges experienced (what has worked and what has not). The question determines whether the team/s are well structured or not?)*

60.	Are there different roles for team members within this team? *Probe for challenges experienced in assigning roles (what has worked and what has not). The question determines whether the team/s are well structured or not?*
61.	Is the team recognized by the community/department/s/official organizations as a clearly defined team? (*The question determines whether the team/s are well structured or not?)*
62.	Does the team meet regularly to discuss its effectiveness and how it could be improved? (*The question determines whether the team/s are well structured or not?)*
	**Actors and competences**
63.	How well do the disciplinary composition and the competence in the team affect them achieving the essential aspects of their objective? *(Focuses on identification of transdisciplinarity in the team/s.)*
64.	Are the competences of the various disciplines appropriate to the problem and its solution (relevant knowledge, role in the case study,<br>possibilities for implementing results)?<br>(*checks for competences of the different disciplines and whether these competences are relevant to the problem?)*
	**Problem formulation, focus, goals and criteria of success**
65.	How relevant is the initiative to One Health? (*The question focuses on identification of the case study (problem) in terms of One Health strategy*
66.	Is the One Health problem adequately translated into scientific questions? Is the current state of knowledge taken into consideration, what is innovative in relation to this state of knowledge *(i.e. checks the scientific questions raised, as well as probes for contribution to new knowledge)*
67.	Do the methods envisioned, the interfaces of transdisciplinary collaboration, the form of integration in practice, and the outcome of the case study fit the solution strategy sought for in One Health? (*checks for One Health objectives in transdisciplinarity and for knowledge integration)*
68.	What are the interdisciplinarity publications already published and yet to be published (i.e. *assess the ties among publications (as outcomes of One Health initiatives) that represent connections between authors, organizations and journals*

End of interview
