# Supplementary material for: A One Health Evaluation of the Southern African Centre for Infectious Disease Surveillance
Source: Front Vet Sci. 2018 Mar 16;5:33. doi: 10.3389/fvets.2018.00033 (PMC5864892; doi:10.3389/fvets.2018.00033)
Supplement: Supplementary file 2 [file Table_2.PDF]

SACIDS One Health Evaluation/Individual questionnaires

Thank you for your interest in our study. We are a research team from the Royal Veterinary College (http://www.rvc.ac.uk/research) in the United Kingdom who works to investigate the One Health Initiatives put in place by SACIDS over the past eight years. This study aims to evaluate and measure the “One Health-ness” of SACIDS capacity building in the surveillance of infectious diseases, using a framework developed by the "Network for Evaluation of One Health" group of experts (http://neoh.onehealthglobal.net/). We will ask you questions regarding SACIDS internal functioning, as well as ask about your view on your methods of working within SACIDS activities.

Assessing the efficiency of these One-Health initiatives is essential to help SACIDS gain efficacy in One Health capacity building, achieve the best results possible, and evolve in future years. Moreover, evaluating One Health initiatives is something new that will need standardised methods in the future. Our study also aims to test and improve the design of a protocol build specifically to evaluate One-Health projects like SACIDS.

It is entirely your choice to decide whether or not you wish to participate to our study.

The information you give us is completely confidential, and the survey is anonymous. Your responses will only be used for research purposes.

We would very much appreciate your help and collaboration in this project. Your participation is important to the success of the study but it is entirely voluntary and unwillingness to participate will not preclude you from access to the results of the research. We believe the results of this project would be useful for you and we would be happy to communicate the results, as well as the scores obtained from the group interviews, when the study is completed.

Completing the survey should take approximately half an hour. If you have any questions regarding this survey or the study in general please contact Marie Hanin (mhanin5@rvc.ac.uk) or Barbara Haesler (bhaesler@rvc.ac.uk).

This study has been approved by the Social Sciences Research Ethical Review Board of the Royal Veterinary College (URN SR2017 - 1002)

* Required

1. **Email address** *

____

Network for Evaluation of One Health

2. **I consent to taking part in this survey.** *

Mark only one oval.

- ( ) Yes
- ( ) No *After the last question in this section, stop filling out this form.*

General Information

3. **For which SACIDS country do you work?** *
Mark only one oval.

- Democratic Republic of Congo
- Mozambique
- South Africa
- Tanzania
- Zambia
- Other: ____________

4. **Name of your Institution** *

____

5. **Gender** *
Mark only one oval.

- Male
- Female
- Prefer not to say

Professional Background

6. **What is your working experience in projects contributing to One Health?** *
Mark only one oval.

- No experience
- 1-5 years
- 6-10 years
- more than 10 years

7. **Which of the following best describes your current role? Please select one.** *
Mark only one oval.

- Upper management
- Middle Management
- Junior Management
- Senior researcher
- Junior researcher
- Self-employed/Partner
- Administrative Staff
- Support Staff
- Trained Professional
- Temporary Employee
- Undergraduate student
- Postgraduate student
- Other: ____________

8. **What is your highest degree obtained?** *
Mark only one oval.

- Some high school, no diploma
- High school graduate, diploma or the equivalent
- Some college credit, no degree
- Trade/technical/vocational training
- Associate degree
- Bachelor’s degree
- Master’s degree
- Professional degree
- Doctorate degree
- Other: ______________________

9. **What is/are your field(s) of experience? Tick as many as apply.** *
Check all that apply.

- [ ] Human medicine
- [ ] Veterinary medicine
- [ ] Statistics
- [ ] Epidemiology
- [ ] Environmental Science
- [ ] Social Sciences
- [ ] Economics
- [ ] Biological Sciences
- [ ] Natural Sciences
- [ ] Bioinformatics
- [ ] Molecular biology
- [ ] Other: ______________________

Assessment of One Health learning

Please read the statements below and tick the relevant boxes. Please select one option only for the following questions.

One Health learning: Individual level

There are three main types of learning, as described below. Please consider them and say how often during your SACIDS time you experienced each type of learning.

10. **No learning** *

Only received information that may lead to learning, but learning was not practised.

Mark only one oval.

- Strongly agree
- Agree
- Neither agree nor disagree
- Disagree
- Strongly disagree
- Don't know

11. **Adaptive learning** *

Learning through which the obtained information is used to correct or improve procedures, existing competences, technologies and paradigms without necessarily examining or challenging the underlying beliefs and assumptions of the organisation.

Mark only one oval.

- Strongly agree
- Agree
- Neither agree nor disagree
- Disagree
- Strongly disagree
- Don't know

12. **Generative learning** *

Learning than involved the modification of the organisation's underlying norms, policies, and objectives that made me able to see beyond the situation and questioning operating norms.

Mark only one oval.

- Strongly agree
- Agree
- Neither agree nor disagree
- Disagree
- Strongly disagree
- Don't know

One Health learning: Team level

At the team level, team-players can share the knowledge better and include more people in the learning process.
Please consider team learning processes and say how often during your SACIDS time you experienced it, in the situations presented below.

13. **The team(s) only meet(s) and exchange information for mere reporting purpose with no goal to support learning.** *

Mark only one oval.

- Strongly agree
- Agree
- Neither agree nor disagree
- Disagree
- Strongly disagree
- Don't know

14. **In the team(s), different views are presented and defended and there is a search for the best view to support decisions that must be made at this time.** *

Mark only one oval.

- Strongly agree
- Agree
- Neither agree nor disagree
- Disagree
- Strongly disagree
- Don't know

15. **In the team(s), complex issues are explored by presenting different views as a mean towards discovering a new view. The assumptions the team(s) members have are presented and examined.** *

Mark only one oval.

- Strongly agree
- Agree
- Neither agree nor disagree
- Disagree
- Strongly disagree
- Don't know

One Health learning: Organisational level

Organisations such as SACIDS can create conditions that may significantly influence the individual learning of people within the organisation. Please consider the following statements and say how often during your SACIDS time you felt the organisation provided sufficient information and knowledge.

16. **The existing information and knowledge that is circulating in SACIDS is collected.** *

Mark only one oval.

- Strongly agree
- Agree
- Neither agree nor disagree
- Disagree
- Strongly disagree
- Don't know

17. **The existing information and knowledge that is circulating in SACIDS is stored.** *
Mark only one oval.

- Strongly agree
- Agree
- Neither agree nor disagree
- Disagree
- Strongly disagree
- Don't know

18. **The existing information and knowledge in SACIDS is made available to teams and individuals in various ways.** *
Mark only one oval.

- Strongly agree
- Agree
- Neither agree nor disagree
- Disagree
- Strongly disagree
- Don't know

One Health learning: Direct environment

The direct environment of SACIDS consists of all the external entities with which SACIDS interacts such as governments, NGOs, institutions, internationl partners etc. Please consider and say how often SACIDS' direct environment supported individual learning.

19. **The direct environment of SACIDS was supportive for adaptive learning - learning that focuses on correcting or improving existing procedures, competences and technologies.** *
Mark only one oval.

- Strongly agree
- Agree
- Neither agree nor disagree
- Disagree
- Strongly disagree
- Don't know

20. **The direct environment of SACIDS was supportive for transformative learning - learning that focuses on questioning the existing norms and that encourages to see beyond the existing situation.** *
Mark only one oval.

- Strongly agree
- Agree
- Neither agree nor disagree
- Disagree
- Strongly disagree
- Don't know

One Health learning: General environment

The general environment of SACIDS consists of non-specific elements of the organisation's surroundings that might affect its learning, such as economics, technology, socio-cultural aspects etc. Please consider and say how often SACIDS' general environment supported individual learning.

21. **The general environment of SACIDS was supportive for adaptive learning - learning that focuses on correcting or improving existing procedures, competences and technologies.** *

Mark only one oval.

- Strongly agree
- Agree
- Neither agree nor disagree
- Disagree
- Strongly disagree
- Don't know

22. **The general environment of SACIDS was supportive for transformative learning - learning that focuses on questioning the existing norms and that encourages to see beyond the existing situation.** *

Mark only one oval.

- Strongly agree
- Agree
- Neither agree nor disagree
- Disagree
- Strongly disagree
- Don't know

23. **How often do you use the following media to enhance your knowledge through individual learning?** *

Mark only one oval per row.

	Every day	Weekly	Monthly	2-3 times of year	Once a year	Less than once a year	Don't know/Not applicable
Internet access to information in my discipline(s) (excluding scientific journals)	⬭	⬭	⬭	⬭	⬭	⬭	⬭
Internet access to information from other disciplines (excluding scientific journals)	⬭	⬭	⬭	⬭	⬭	⬭	⬭
Peer-reviewed article(s) from national journals	⬭	⬭	⬭	⬭	⬭	⬭	⬭
Peer-reviewed article(s) from international journals	⬭	⬭	⬭	⬭	⬭	⬭	⬭
Non-peer-reviewed article(s) from national journals/magazines/books	⬭	⬭	⬭	⬭	⬭	⬭	⬭
Non-peer-reviewed article(s) from international journals/magazines/books	⬭	⬭	⬭	⬭	⬭	⬭	⬭
Conference(s) attendance	⬭	⬭	⬭	⬭	⬭	⬭	⬭
Short course(s) at my institution	⬭	⬭	⬭	⬭	⬭	⬭	⬭
Short course(s) online	⬭	⬭	⬭	⬭	⬭	⬭	⬭
Short course(s) at institutions other than mine	⬭	⬭	⬭	⬭	⬭	⬭	⬭
Discussions with colleagues from my disciplines	⬭	⬭	⬭	⬭	⬭	⬭	⬭
Discussions with colleagues from other disciplines	⬭	⬭	⬭	⬭	⬭	⬭	⬭

Interdisciplinary competence scale

- Please read each question carefully and and respond to the items as accurately as you can.
- Do not spend too long thinking about your responses to an item, usually your first reaction is the best one.
- Most statements ask you to indicate the degree or extent of your view by marking the right box on a predetermined scale which best reflects your opinion. Always mark one box for each question or statement.

24. **Interdisciplinary skills : To what extent do you agree with the following?** *

Please indicate the extent to which you either agree or disagree with each statement.

Mark only one oval per row.

	Strongly disagree	Disagree	Neither agree nor disagree	Agree	Strongly agree	Don't know/Not applicable
We all influence each other						
I value reading about topics outside my discipline						
I enjoy thinking about how different fields approach the same problem in different ways						
Not all One Heath problems have purely technical solutions						
In solving One Health problems I often seek information from experts in other academic fields						
Given knowledge and ideas from different fields, I can figure out what is appropriate for solving a One Health problem						
I see connections between ideas in my discipline and ideas in other fields including humanities and social sciences						
I can take ideas from outside my discipline and synthesize them in ways that help me better understand						
I can use what I have used in one field in another setting						

25. **Reflective behaviour: To what extent do you agree with the following?** *

Please indicate the extent to which you either agree or disagree with each statement.

Mark only one oval per row.

	Strongly disagree	Disagree	Neither agree nor disagree	Agree	Strongly agree	Don't know/Non applicable
I often step back and reflect on what I am thinking to determine whether I might be missing something						
I frequently stop to think about where I might be going wrong or right with a problem solution						

26. **Recognising Disciplinary Perspectives: To what extent do you agree with the following?** *

Please indicate the extent to which you either agree or disagree with each statement.
Mark only one oval per row.

	Strongly disagree	Disagree	Neither agree nor disagree	Agree	Strongly agree	Don't know/Non applicable
If asked, I could identify the kinds of knowledge and ideas that are distinctive to different fields of study	◯	◯	◯	◯	◯	◯
I recognize the kinds of evidence that different fields of study rely on	◯	◯	◯	◯	◯	◯
I'm good at figuring out what experts in different fields have missed in explaining a problem or a solution	◯	◯	◯	◯	◯	◯

27. **Comments**

Do you have additional comments you would like to make in relation to interdisciplinary competence covered in this survey?

____

____

____

____

____

Assessment of One Health thinking

Dimensions level

28. **Which dimensions are considered in capacity building for One Health surveillance for infectious diseases in SACIDS and what is their importance? Each dimension can have different scales, as described in the following questions.** *

Please tick the appropriate box for each dimension.
Mark only one oval per row.

	Not considered (Not important at all)	Passive recognition (low importance)	Considered (Slighlty important)	Weak involvement (moderately important)	Strong involvement (very important)	Essential (extremely important)	Don't know
Spatial	◯	◯	◯	◯	◯	◯	◯
Temporal	◯	◯	◯	◯	◯	◯	◯
Network	◯	◯	◯	◯	◯	◯	◯
Institutional	◯	◯	◯	◯	◯	◯	◯
Economics	◯	◯	◯	◯	◯	◯	◯
Knowledge	◯	◯	◯	◯	◯	◯	◯
Jurisdictional	◯	◯	◯	◯	◯	◯	◯
Religion	◯	◯	◯	◯	◯	◯	◯
Leadership	◯	◯	◯	◯	◯	◯	◯

29. **If the dimension was excluded, how strongly would this affect the outcomes of the capacity building for One Health surveillance for infectious diseases in SACIDS?** *
Please tick the appropriate box for each dimension.
Mark only one oval per row.

	No effect	Slight effect	Some effect	Moderate effect	Strong effect	Very strong effect	Don't know
Spatial	◯	◯	◯	◯	◯	◯	◯
Temporal	◯	◯	◯	◯	◯	◯	◯
Network	◯	◯	◯	◯	◯	◯	◯
Institutional	◯	◯	◯	◯	◯	◯	◯
Economics	◯	◯	◯	◯	◯	◯	◯
Knowledge	◯	◯	◯	◯	◯	◯	◯
Jurisdictional	◯	◯	◯	◯	◯	◯	◯
Religion	◯	◯	◯	◯	◯	◯	◯
Leadership	◯	◯	◯	◯	◯	◯	◯

30. **How strongly does the dimension affect the capacity building for One Health surveillance for infectious diseases in SACIDS?** *
Please tick the appropriate box for each dimension.
Mark only one oval per row.

	No effect	Slight effect	Some effect	Moderate effect	Strong effect	Very strong effect	Don't know
Spatial	◯	◯	◯	◯	◯	◯	◯
Temporal	◯	◯	◯	◯	◯	◯	◯
Network	◯	◯	◯	◯	◯	◯	◯
Institutional	◯	◯	◯	◯	◯	◯	◯
Economics	◯	◯	◯	◯	◯	◯	◯
Knowledge	◯	◯	◯	◯	◯	◯	◯
Jurisdictional	◯	◯	◯	◯	◯	◯	◯
Religion	◯	◯	◯	◯	◯	◯	◯
Leadership	◯	◯	◯	◯	◯	◯	◯

Scales level

For each dimension selected above, which components or scales are considered by SACIDS for the capacity building of One-Health surveillance of infectious diseases? Tick as many as apply.

31. **Spatial** *
For the spatial dimension cited above, which geographical level(s) of analysis (or scales) is/are considered by SACIDS for the capacity building for One Health surveillance of infectious diseases? Tick as many as apply.
Check all that apply.

- [ ] Farm level
- [ ] District level
- [ ] National level
- [ ] International level
- [ ] Global level
- [ ] Don't know
- [ ] Other: ____________

32. **Temporal** *

For the temporal dimension cited above, which time scale(s) is/are considered by SACIDS for the capacity building for One Health surveillance of infectious diseases? Tick as many as apply.
Check all that apply.

- [ ] Days
- [ ] Weeks
- [ ] Months
- [ ] Years
- [ ] Decades
- [ ] Don't know
- [ ] Other: ______________________________

33. **Network** *

For the network dimension cited above and within SACIDS initiative, at which level(s) can people build a network with people from other backgrounds or levels? Tick as many as apply.
Check all that apply.

- [ ] Individual level
- [ ] Institutional level
- [ ] Sectorial level
- [ ] Ministerial level
- [ ] International Institutions level
- [ ] Don't know
- [ ] Other: ______________________________

34. **Institutional** *

For the institutional dimension cited above, which external group(s) do people from SACIDS interact and connect with? Tick as many as apply.
Check all that apply.

- [ ] Individuals
- [ ] NGOs
- [ ] Governments
- [ ] Research institutes
- [ ] International Institutes
- [ ] Don't know
- [ ] Other: ______________________________

35. **Economics** *

For the economic dimension cited above, at which level(s) does SACIDS get involved for the capacity building for One Health surveillance of infectious diseases? Tick as many as apply.
Check all that apply.

- [ ] Farm or household level
- [ ] Institutional level
- [ ] Sectoral level
- [ ] National level
- [ ] International level
- [ ] Don't know
- [ ] Other: ______________________

36. **Knowledge** *

For the knowledge dimension cited above, which scale(s) of knowledge (individual or collective) is/are considered by SACIDS for the capacity building for One Health surveillance of infectious diseases? Tick as many as apply.
Check all that apply.

- [ ] Individual level
- [ ] Team level
- [ ] Institutional level
- [ ] Sectorial level
- [ ] Ministerial level
- [ ] National level
- [ ] Regional level
- [ ] Don't know
- [ ] Other: ______________________

37. **Jurisdictional** *

For the jurisdictional dimension cited above, which level(s) (or scales) of regulations from Standard Operating Procedures is/are considered by SACIDS for the capacity building for One Health surveillance of infectious diseases? Tick as many as apply.
Check all that apply.

- [ ] Local laws
- [ ] National laws
- [ ] Regional laws
- [ ] International bilateral agreement
- [ ] International institutions recommendations
- [ ] Don't know
- [ ] Other: ______________________

38. **Religion** *

For the religious dimension cited above, which level(s) (or scales) of faith and values is/are considered by SACIDS for the capacity building for One Health surveillance of infectious diseases? Tick as many as apply.

Check all that apply.

- [ ] Personal
- [ ] Group values
- [ ] Ethnic values
- [ ] Religious values
- [ ] Global values
- [ ] Don't know
- [ ] Other: ____________________

39. **Leadership** *

For the leadership dimension cited above, which level(s) (or scales) of leadership is/are considered by SACIDS for the capacity building for One Health surveillance of infectious diseases? Tick as many as apply.

Check all that apply.

- [ ] Individual
- [ ] Groups
- [ ] Institutions
- [ ] Disciplines
- [ ] Ministries
- [ ] Don't know
- [ ] Other: ____________________

One Health capacity builing

40. **What features of the system are targeted by the capacity building for One Health surveillance for infectious diseases in SACIDS.** *

Please select one.

Mark only one oval.

- ( ) Singular events - Reactions to events
- ( ) Patterns - let us understand reality at a deeper level, i.e.trends, changes over time to which we can adapt
- ( ) Structural - thinking at structural level means thinking in terms of causal connections
- ( ) Don't know
- ( ) Other: ____________________

41. **What is the principal underlying driver or desire for capacity building for One Health surveillance for infectious diseases in SACIDS.** *

Please select one.
Mark only one oval.

- Correcting damage
- Containing damage
- Preventing damage
- Avoiding the problem
- Redirecting the problem
- Modifying the socio-ecological system
- Don't know
- Other: ____________________

42. **Sustainability relies on three pillars of society, environment and economy. One Health integrates the aspects of human, animal, plant and environmental health. Please tick which pillars and aspects are considered for capacity building for One Health surveillance for infectious diseases in SACIDS.** *

Tick as many as relevant.
Check all that apply.

- [ ] Pillar society
- [ ] Pillar economy
- [ ] Pillar environment
- [ ] Human health
- [ ] Animal health
- [ ] Plant health
- [ ] Environmental health

Data collection, Data Analysis and collation of the information

43. **In my country there is sufficient capacity for integrated planning of disease surveillance.** *

With planning we mean elaboration of the surveillance design, including consideration of feasibility/practical factors. Please select one.
Mark only one oval.

- Strongly agree
- Agree
- Neither agree nor disagree
- Disagree
- Strongly disagree
- Don't know

44. **Please explain your answer to the preceding question.**

____
____
____
____
____

45. **In my country there is sufficient capacity for One Health disease surveillance data collection.** *

Please select one.

Mark only one oval.

- Strongly agree
- Agree
- Neither agree or disagree
- Disagree
- Strongly disagree
- Don't know

46. **Please explain your answer to the preceding question.**

47. **I work closely with colleagues from different fields to develop integrated data collection plans for disease surveillance.** *

E.g.: Simultaneous collection of human and animal health data in a community, matching sampling protocols, shared databases... Please select one.

Mark only one oval.

- Strongly agree
- Agree
- Neither agree nor disagree
- Disagree
- Strongly disagree
- Don't know
- Other:

48. **Please explain your answer to the preceding question.**

49. **I feel the results obtained by research teams are adequately communicated to other people who are not part of SACIDS.** *
Please select one.
Mark only one oval.

- Strongly agree
- Agree
- Neither agree nor disagree
- Disagree
- Strongly disagree
- Don't know
- Other: ____________________

50. **Please explain your answer to the preceding question**

____
____
____
____
____

51. **I feel the results obtained by research teams are adequately communicated to other people working in other SACIDS institutions.** *
Please select one.
Mark only one oval.

- Strongly agree
- Agree
- Neither agree nor disagree
- Disagree
- Strongly disagree
- Don't know
- Other: ____________________

52. **Please explain your answer to the preceding question**

____
____
____
____
____

Laboratory capacity building

53. **Do you work in a laboratory setting?** *
Mark only one oval.

- Yes
- No. *Skip to question 67.*

Laboratory capacity building

54. **When I have doubts on a diagnostic procedure, I can contact people from other SACIDS institutions/laboratories and exchange experiences with them or discuss questions.** *

Please select one.
Mark only one oval.

- Strongly agree
- Agree
- Neither agree nor disagree
- Disagree
- Strongly disagree
- Don't know

55. **Please explain your answer to the preceding question**

56. **When I have doubts on a diagnostic procedure, I have the possibility to send the samples to other SACIDS institutions/laboratories for advice.** *

Please select one.
Mark only one oval.

- Strongly agree
- Agree
- Neither agree nor disagree
- Disagree
- Strongly disagree
- Don't know

57. **Please explain your answer to the preceding question**

58. **I feel the laboratory infrastructures and capacities for diagnostic procedures for One Health surveillance are:** *

Please select one

Mark only one oval.

- Very poor
- Poor
- Adequate
- Good
- Very Good
- Don't know

59. **Please explain your answer to the preceding question**

60. **There is good and sufficient communication between SACIDS laboratories within my country to rapidly alert on disease outbreaks** *

Please select one

Mark only one oval.

- Strongly agree
- Agree
- Neither agree nor disagree
- Disagree
- Strongly disagree
- Don't know

61. **Please explain your answer to the preceding question**

62. **There is good and sufficient communication between SACIDS laboratories from different countries to rapidly alert and inform on disease outbreaks** *

Please select one.
Mark only one oval.

- Strongly agree
- Agree
- Neither agree nor disagree
- Disagree
- Strongly disagree
- Don't know

63. **Please explain your answer to the preceding question**

64. **How would you qualify your skills gained with SACIDS in diagnostic procedures in relation to your work contribution in disease surveillance?** *

Please select one.
Mark only one oval.

- Very poor
- Poor
- Adequate
- Good
- Very Good
- Non applicable

65. **Please explain your answer to the preceding question**

66. **Since 2008, and under SACIDS initiative, I feel the infrastructures established for disease surveillance:** *
Please select one.
Mark only one oval.

- Have been largely improved
- Have been slightly improved
- Stayed the same
- Are slightly less functional
- Are extremely less functional
- Don't know

67. **Please explain your answer to the preceding question**

Data Analysis capacity building

68. **Do you work as a data analyst?** *
Mark only one oval.

- Yes
- No *Skip to question 81.*

Data Analysis capacity building

69. **When I have doubts on a surveillance data analysis procedure, I can contact people from other SACIDS institutions/laboratories and exchange experiences with them or discuss some questions.** *
Please select one.
Mark only one oval.

- Strongly agree
- Agree
- Neither agree nor disagree
- Disagree
- Strongly disagree
- Don't know

70. **Please explain your answer to the preceding question**

71. **When I have doubts on a surveillance data analysis procedure, I have the possibility to send the data sheet to other SACIDS institutions/laboratories for advice.** *

Please select one.
Mark only one oval.

- Strongly agree
- Agree
- Neither agree nor disagree
- Disagree
- Strongly disagree
- Don't know

72. **Please explain your answer to the preceding question**

73. **I feel the bioinformatics and technological infrastructures and capacities for diagnostic procedures for One Health surveillance are:** *

Please select one.
Mark only one oval.

- Very poor
- Poor
- Adequate
- Good
- Very Good
- Don't know

74. **Which professions are commonly involved in the data analysis process for surveillance of infectious diseases in your country?** *

Tick as many as apply.
Check all that apply.

- [ ] Human medicine
- [ ] Veterinary medicine
- [ ] Data analysis
- [ ] Environmental sciences
- [ ] Social sciences
- [ ] Economics
- [ ] Biological sciences
- [ ] Natural sciences
- [ ] Don't know
- [ ] Other: ____________

75. **Please explain your answer to the preceding question**

76. **There is good and sufficient communication between SACIDS data analysts for surveillance of infectious diseases within my country.** *

Please select one.
Mark only one oval.

- Strongly agree
- Agree
- Neither agree nor disagree
- Disagree
- Strongly disagree
- Don't know

77. **Please explain your answer to the preceding question.**

78. **There is good and sufficient communication between data analysts for surveillance of infectious diseases from different SACIDS countries.** *

Please select one.
Mark only one oval.

- Strongly agree
- Agree
- Neither agree nor disagree
- Disagree
- Strongly disagree
- Don't know

79. **Please explain your answer to the preceding question**

80. **How would you qualify your skills gained with SACIDS in data analysis in relation to your everyday work?** *

Please select one.
Mark only one oval.

- Very poor
- Poor
- Adequate
- Good
- Very Good
- Non applicable

81. **Please explain your answer to the preceding question**

Final Comments

82. **In your opinion what are the top three SACIDS achievements over the past eight years?** *

83. **In your opinion what are the top three SACIDS disappointments or failures over the past eight years?** *

84. **Please add any further comments or concerns you have regarding this questionnaire.**

Thank you very much for your participation !

If you have any questions regarding this study, please contact Marie Hanin from the Royal Veterinary College at mhanin5@rvc.ac.uk.

A copy of your responses will be emailed to the address you provided

Powered by
Google Forms
